# Performance analysis of UAV-assisted coverage for cell edge

**DOI:** 10.1371/journal.pone.0346901

**Published:** 2026-05-14

**Authors:** Huakui Sun, Yang Zhou

**Affiliations:** 1 School of Airspace Science and Engineering, Shandong University, Shandong, China; 2 School of Electronics and Information, Northwestern Polytechnical University, Xi’an, China; Northwestern Polytechnical University, CHINA

## Abstract

Unmanned aerial vehicles (UAVs), with their rapid and flexible deployment capabilities, have emerged as an effective solution for providing emergency wireless connectivity in scenarios where traditional ground base stations (GBSs) cannot offer reliable communication links. To address the weak coverage experienced by cell-edge users associated with a GBS, this paper investigates a UAV-assisted surround-enhancement coverage strategy. In the proposed approach, the original cell is partitioned into two service regions, where the GBS and UAV individually support users within their designated coverage areas. To improve spectrum utilization efficiency, we analyze the mutual interference between the UAV and GBS under a spectrum-sharing framework. Meanwhile, the user distribution within the cell is modeled as a homogeneous Poisson point process, and both the GBS and UAV randomly allocate channels to their associated users. Analytical results demonstrate that the UAV-assisted scheme exhibits an optimal surround radius and flight altitude, and that a reasonable division of coverage regions between the GBS and UAV can fully leverage their respective coverage capabilities.

## Introduction

With the evolution of communication networks toward an integrated air-ground architecture, the role of unmanned aerial vehicle (UAV) networks has become increasingly prominent. UAV-assisted cellular communications, as a practical implementation of UAV networking, have emerged as an important research direction for sixth generation mobile networks (6G) [1,2]. When deployed as aerial base stations, UAVs can flexibly adjust their altitude and position, thereby providing high system throughput and reliable line-of-sight (LoS) coverage for ground user equipment [3,4]. Such three-dimensional (3D) deployment helps mitigate blockage effects, as the link distance between the UAV and the user is determined by the UAV’s 3D location. Therefore, analyzing the performance of UAVs coexisting with ground base stations (GBSs) is essential for the effective design of integrated air-ground heterogeneous networks.

Providing effective coverage while maintaining efficient spectrum utilization introduces challenges in UAV-GBS coexistence. Efficient resource allocation and cooperative communication strategies are essential to enhance link quality in heterogeneous networks [5,6]. For instance, aerial downlink cooperative communication schemes have been proposed, in which UAVs act as aerial relays to forward ground user signals to central processors [7]. Coordinated multipoint transmission techniques can further mitigate downlink interference and improve connectivity between UAVs and GBSs [8,9]. These strategies demonstrate the potential of UAV-GBS cooperation to improve both uplink and downlink performance in integrated air-ground networks. Cell-edge coverage remains a particularly challenging problem. Users at the edge experience weaker signals from GBSs and are more susceptible to interference from neighboring cells. In this context, low-altitude UAVs can provide flexible coverage extensions, offering high-speed links in dense or hotspot areas [10,11]. By dynamically adjusting their position, UAVs can establish direct wireless links to edge users, significantly improving coverage compared to fixed GBS deployments. This flexible deployment also enables more effective spectrum utilization, ensuring connectivity in regions where GBSs alone may not provide adequate service.

Accurate modeling of UAV and user distributions is essential for reliable performance analysis. For user-centered communication scenarios, [12] modeled UAVs at a fixed flight height and users as a poisson cluster processes (PCPs) centered on the UAV’s ground projection, analyzing the impact of UAV altitude on coverage and path loss. Using poisson point processes (PPP), [13] represented UAVs with homogeneous PPP and GBSs with non-homogeneous PPP to capture their distinct service scenarios and coverage performance. Considering the altitude advantage of UAVs, [14] modeled drones as three-dimensional homogeneous PPP (HPPP) to study spectrum sharing in UAV small cells and determine their optimal density, while [15] analyzed successful transmission probability and spectral efficiency in multi-layer aerial networks. For finite UAV networks, [16] used binomial point processes (BPPs) to model UAV positions and derived downlink coverage probability. Although 3D point processes capture UAV altitude randomness, they complicate calculations when the height range is limited. Therefore, most existing works adopt 2D point processes, with height parameters introduced separately to reflect UAV flight characteristics [17]. In specific deployment scenarios, non-PPPPs are more appropriate. For example, BPPs can model limited-scale UAV deployment in hotspots [18–20], while poisson hole processes can represent UAVs with exclusion zones to avoid interference [21]. User distributions are often modeled using PCPs to reflect clustering around base stations. To manage collisions and interference, repulsive point processes such as Matern hard-core processes have also been applied [22,23]. However, while stochastic geometry enables closed-form performance expressions, the use of specialized point processes often complicates derivations, and few studies have incorporated them into UAV-assisted network analyses [24]. In cases without explicit distance constraints, UAVs are typically modeled using HPPPs.

Despite these advances, several important issues remain insufficiently addressed. Existing works primarily focus on static UAV hovering or trajectory optimization, while UAVs performing circular or ring-shaped motion to assist cell-edge coverage have received limited analytical investigation. In addition, most current analyses approximate UAV distributions as random point processes, which do not capture the deterministic geometric structure of UAV edge-surround coverage, nor the associated variations in user incident angles. Furthermore, joint analysis of LoS/non-line-of-sight (NLoS) propagation, external interference, and collaborative interference from central base stations has not been fully integrated into a unified theoretical framework. These gaps motivate a rigorous stochastic-geometry-based analysis tailored to UAV-assisted edge coverage.

To address the above limitations, we develops a comprehensive analytical framework for a heterogeneous UAV-assisted cellular network, where a UAV performs circular surround coverage at the cell edge. The main contributions of this work are summarized as follows:

- To address the weak coverage issue of cell-edge users served by ground base stations (GBSs), this paper proposes a targeted UAV circling coverage enhancement scheme. The original cell is divided into two non-overlapping service regions, where GBSs and UAVs serve users in their respective coverage areas independently. This partitioned coverage strategy reduces the service range of both access points, eliminates coverage blind spots for cell-edge users, and improves edge signal coverage quality.
- A comprehensive interference analysis framework is developed for spectrum sharing between UAVs and ground base stations (GBSs). To fully utilize scarce spectrum resources, we analyze the co-channel interference characteristics and quantify its impact on the communication performance of both access points. This work provides a theoretical foundation for efficient spectrum allocation and interference coordination in air-ground integrated networks.
- A systematic performance analysis model for air-ground collaborative coverage is established based on HPPP. Cell user distribution follows HPPP, and a random channel allocation scheme is adopted to match practical scenarios. Via theoretical derivation, the optimal UAV circling radius and flight altitude are obtained, with the quantitative link between rational cell partition and coverage performance clarified. Results prove that optimal UAV deployment and reasonable area division can maximize the coverage merits of UAVs and GBSs.

The rest of this article is organized as follows. In the System Model section, the UAVS edge-assisted cellular network model is described, in which the channel models of different types of transmission links are introduced in detail. In the Performance Analysis section, the communication performance of different types of base stations is analyzed, and the interference probability and coverage performance between each other are solved. The numerical simulation results and theoretical results are discussed in the Simulation Results section. Finally, the Conclusion section presents the conclusion of the paper.

## System model

### Network model

**Fig 1:**
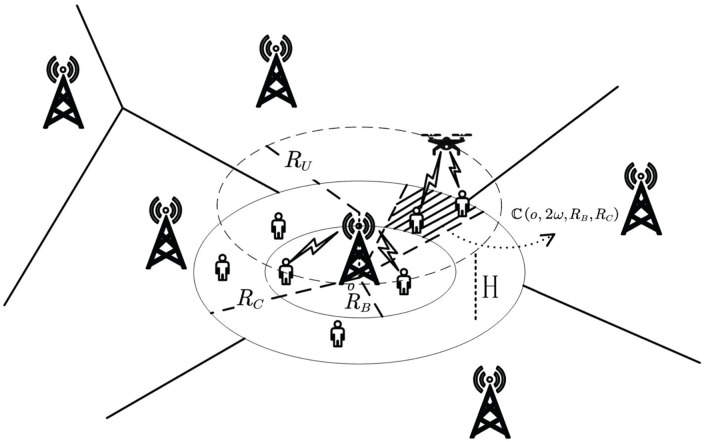
The air-and-ground integrated network network.

In this paper, we consider a GBS network distributed over a two-dimensional plane, as shown in Fig 1. To facilitate the analysis of a target cell, the GBS of the target cell is located at the coordinate origin *o* with a cell radius *R*_*C*_, and the corresponding cell area is denoted as 𝔹. To capture the widespread and random interference from external base stations, the interfering GBSs are modeled as a HPPP ${𝛷}_{G}$ with density $\lambda_{G}$ distributed over ${\mathbb{R}}^{2}⧵𝔹$. Denoting the position of the *i*-th GBS as *z*_*i*_, the set of interfering base stations is ${𝛷}_{G}=\{z_{i},i\in {\mathbb{N}}^{+}\}\subset {\mathbb{R}}^{2}⧵𝔹$.

Users at the cell edge suffer from poor signal coverage due to signal attenuation and obstacle occlusion, which has become a performance bottleneck of cellular networks. To address this issue, we deploy UAV-mounted aerial BSs to enhance the coverage at the cell edge, as shown in Fig 1. Specifically, GBSs provide communication services for proximal users, while UAVs serve edge users. They collaborate to improve network performance. Its advantages are as follows: First, UAVs are deployed at the cell edge to shorten the distance between transceivers, thereby enhancing the signal coverage strength. Second, the high flight altitude of UAVs can increase the probability of line-of-sight (LoS) propagation, thus improving the information transmission rate. Third, UAVs can be dynamically deployed on demand to avoid obstacle occlusion and further optimize network performance. Therefore, the air-and-ground integrated network has significant advantages over the purely terrestrial network.

To enhance the coverage at the cell edge, a UAV flies above the target base station at a fixed altitude *H* along a circular trajectory with radius *R*_*U*_, moving at a uniform speed to serve users in the cell-edge area. The UAV shares the same spectrum resources with the GBS. Accordingly, the cell area can be divided into two regions: the reduced GBS coverage radius *R*_*B*_, corresponding to the inner circular area ${𝔹}_{B}\subset 𝔹$, and the remaining ring-shaped area ${𝔹}_{U}=𝔹⧵{𝔹}_{B}$ served by the UAV.

Considering the UAV’s circular motion, it only serves users within its instantaneous coverage area at any given time. Let the line connecting the UAV’s ground projection and the macro base station serve as the axis of symmetry. The UAV instantaneously covers users in the arc-shaped area $\mathbb{C}(o,2\omega ,R_{B},R_{C})$, with center *o*, central angle 2ω, and radii ranging from *R*_*B*_ to *R*_*C*_. As the UAV moves periodically ω takes the range ω∈[0,π].

In this paper, the user distribution within the cell is modeled as a HPPP ${𝛷}_{UE}$ with density $\lambda_{UE}$. As a result, the number of users in the cell is random. This paper adopts the Orthogonal Frequency Division Multiple Access (OFDMA) technology, where each user is assigned an orthogonal subchannel. As a result, intra-cell interference is completely eliminated within a single cell, while inter-cell interference arises among different cells due to spectrum reuse. Such inter-cell interference is fully incorporated into the performance analysis to ensure the accuracy and practicality of the theoretical results. To provide coverage for multiple users at the same time, the frequency resources are divided between the GBS and the UAV. Each user within their respective coverage areas is randomly assigned a channel.

Due to the properties of HPPP, the user distribution remains uniform. Under this uniform distribution assumption, the coverage performance of the entire network can be evaluated by analyzing the coverage condition of the UAV at a given time. In this analysis, the UAV is treated as stationary. In practical deployment, the UAV’s circular motion affects which users are served at each moment. This effect does not change the theoretical performance analysis.analysis.

### Channel model

In this paper, both large-scale fading and small-scale fading are considered in the channel characteristics. LoS and NLoS transmissions exhibit different propagation behaviors. Large-scale fading is caused by path loss, while small-scale fading is modeled as Nakagami-m fading. This framework can describe the characteristics of both LoS and NLoS links uniformly.

If the transmitted signal power is *P*_*t*_, the received signal power under LoS and NLoS conditions is given by:


$$\begin{array}{c} P_{r}=\left\{ \begin{array}{c} \eta_{L}P_{t}l^{-\alpha_{L}}g_{L},\;\;\;\;\;if\;LoS\;channel,\\ \eta_{N}P_{t}l^{-\alpha_{N}}g_{N},\;\;if\;NLoS\;channel, \end{array}\right. \end{array}$$ (1)

Where $\eta_{L}$ and $\eta_{N}$ represent the path loss coefficients when the reference distance of the link is 1 m. *l* is the distance between the user and the serving base station. $\alpha_{L}$ and $\alpha_{N}$ ($2\leq \alpha_{L}<\alpha_{N}$) are the path loss exponents for LoS and NLoS links respectively and *g*_*L*_ and *g*_*N*_ denote the sma*l*l-scale channel power gains.

For small-scale fading channels, LoS links are modeled using Nakagami-m fading, while NLoS links are modeled using Rayleigh fading. Under the Nakagami-m fading assumption, *g*_*L*_ follows a gamma distribution with shape and scale parameters *m*. For NLoS links, Rayleigh fading is equivalent to a Nakagami-m distribution with *m* = 1. *g*_*N*_ of NLoS link is distributed exponentially with unit mean.

The properties of the random variable *m* play a crucial role in the theoretical analysis in the following analysis. Its complementary cumulative distribution function (CCDF) is given by:


$$\begin{array}{c} F\left(x\right)=1-e^{-mx}\sum_{k=0}^{m-1}\frac{{\left(mx\right)}^{k}}{k!}. \end{array}$$ (2)

According to the nature of the derivation function, the above equation can be converted to:


$$\begin{array}{c} F\left(x\right)=1-\frac{{\left(-1\right)}^{m-1}m^{m}}{\left(m-1\right)!}\cdot \frac{\partial^{m-1}}{\partial m^{m-1}}\cdot \frac{e^{-mx}}{m}, \end{array}$$ (3)

where *x* appears in the exponent section. When *m* is large, the computation of higher-order derivatives becomes more complex. For the sake of analysis, we adopt the tight upper bound of the CCDF provided by the Alzer lemma, as shown in [25]:


$$\begin{array}{c} F\left(x\right)⩽\sum_{n=1}^{m}C_{n}^{m}{\left(-1\right)}^{n+1}e^{-nDx}, \end{array}$$ (4)

where $D={\left(m!\right)}^{-\frac{1}{m}}m$. This formula can be used to approximate the coverage probability in the following analysis. The equality also holds when *m* = 1. Due to the randomness of small-scale fading, it is necessary to compute the Laplace transform of the interference.

For the UAV, the probability $P_{U,L}\left(r\right)$ that a user under its coverage has a LoS communication link is given in [11]:


$$\begin{array}{c} P_{U,L}\left(r\right)=\frac{1}{1+c\exp\left(-b\left(\varphi \left(\frac{H}{r}\right)-c\right)\right)}, \end{array}$$ (5)

where $\varphi \left(x\right)=\frac{180}{\pi }arctan\left(x\right)$represents the elevation angle of the link. The constant *c* depends on the environment, *H* is the flight height of the UAV, and *r* is the horizontal distance between the UAV and the user. It can be seen from the formula that the UAV’s flight height is positively correlated with the LoS probability of the link. Increasing the UAV flight height helps improve the LoS probability. Correspondingly, the probability of an NLoS link is:


$$\begin{array}{c} P_{U,N}\left(r\right)=1-P_{U,L}\left(r\right). \end{array}$$ (6)

The probabilistic LoS channel is the preferred model for UAV air-ground links, and the core reasons are as follows, which align with the actual propagation characteristics of UAV air-ground channels and the practical needs of network performance analysis and optimization [26]: (1) It accurately depicts the dynamic switching between LoS and NLoS in air-ground propagation. (2) It enables quantitative characterization of channel fading and link quality. (3) It adapts to the 3D dynamic deployment and network optimization of UAVs. (4) It balances model authenticity and analytical solvability.

For the GBS, the probability $P_{G,L}\left(r\right)$that the link between the base station and a user is LoS is given in [27]:


$$\begin{array}{c} P_{G,L}\left(r\right)=e^{-\beta r}, \end{array}$$ (7)

where βis a constant that depends on the geometry and density of buildings, and *r* is the distance between the GBS and the user. Similarly, the NLoS probability of the link between the GBS and the user is:


$$\begin{array}{c} P_{G,N}\left(r\right)=1-P_{G,L}\left(r\right). \end{array}$$ (8)

### SINR model

Considering that the spectrum of the base station and the UAV is divided into *M* parts, the transmission power is equally distributed across the channels. Consequently, all types of interference are reduced due to spectrum allocation. The SINR for a GBS user can thus be expressed as:


$$\begin{array}{cc} SINR_{UE,B}= & \frac{P_{UE,B}/M}{I_{R_{C}}/M+I_{UI}/M+N_{0}/M}\\ = & \frac{P_{UE,B}}{I_{R_{C}}+I_{UI}+N_{0}}, \end{array}$$ (9)

where *P*_*UE*,*B*_ denotes the total received signal power of the GBS user, $I_{R_{C}}$ represents the interference from GBSs outside the radius *R*_*C*_, which is assumed to be NLoS for analytical simplicity. *I*_*UI*_ is the interference from the UAV, and *N*_0_ is the noise power. It can be concluded that under this strategy, frequency division does not affect the users’ received signal quality. However, when the number of users is fixed, using more channels helps reduce mutual interference between the base station and the UAV, allowing the frequency division strategy to provide coverage for more users simultaneously.

In contrast, for a user covered by a UAV at a given time, the SINR is:


$$\begin{array}{c} SINR_{UE,U}=\frac{P_{UE,U}}{I_{R_{C}}+I_{BI}+N_{0}}, \end{array}$$ (10)

where *P*_*UE*,*U*_ denotes the total received signal power of the UAV user, and *I*_*BI*_ represents the interference power from the GBS at the center of the cell. Therefore, the effect of channel division on the received power can be neglected when analyzing the SINR distribution.

### Network throughput and traversal rate

In the proposed network model, users are distributed according to a HPPP with density $\lambda_{UE}$. The GBS at the center of the cell and the edge UAV randomly assign channels to users within their coverage areas. If there are *N* users in an area *S* and the total number of channels is *M*, the number of users assigned channels is min(M,N). Consequently, the average number of users that can be served is E[min(M,N)].

For communication with a fixed data rate, according to Shannon’s formula, the SINR requirement is constant, and whether a user can successfully receive the signal depends on coverage. For a typical SINR threshold *T*, the throughput can be expressed as:


$$\begin{array}{c} 𝒯=P\left(SINR⩾T\right)\log_{2}\left(1+T\right). \end{array}$$ (11)

for *N*_*B*_ users under GBS coverage, the SINR distribution and channel allocation ensure that each user equally shares the total spectrum resources. Therefore, the throughput under GBS coverage can be expressed as:


$$\begin{array}{c} {𝒯}_{B}\;=\;\frac{E\left[\min\;\left(M,N_{B}\right)\right]}{M}P\left(SINR_{UE,B}\;⩾\;\gamma \right)\log_{2}\left(1\;+\;\gamma \right). \end{array}$$ (12)

Similarly, the total throughput of *N*_*U*_ users under UAV coverage is:


$$\begin{array}{c} {𝒯}_{U}\;=\;\frac{E\left[\min\left(M,N_{U}\right)\right]}{M}P\;\left(SINR_{UE,U}\;⩾\;\gamma \right)\log_{2}\;\left(1\;+\;\gamma \right). \end{array}$$ (13)

Thus, for UAV-assisted coverage, the total network throughput is:


$$\begin{array}{c} {𝒯}_{UU}=T_{B}+T_{U}. \end{array}$$ (14)

For comparison, the network throughput without using UAVs is given by:


$$\begin{array}{c} {𝒯}_{NU}\;=\;\frac{E\left[\min\left(M,N_{NU}\right)\right]}{M}P\left(SINR_{UE,NU}\;⩾\;\gamma \right)\log_{2}\left(1\;+\;\gamma \right), \end{array}$$ (15)

where *N*_*NU*_ denotes the number of users without UAV assistance, and *SINR*_*UE*,*NU*_ represents the corresponding SINR.

Traversal rate is another important performance metric. It refers to the maximum rate at which a channel codeword can traverse all fading states, i.e., the highest rate at which the system can transmit correctly over fading channels. The traversal rate ℛ of a communication system under fading channels can be expressed as:


$$\begin{array}{cc} ℛ= & E\left[\log_{2}\left(1+SINR\right)\right]\\ = & \int_{0}^{\infty }P\left(\log_{2}\left(1\;+\;SINR\right)\;⩾\;x\right)dx\\ = & \int_{0}^{\infty }P\left(SINR⩾2^{x}-1\right)dx\\ = & \frac{1}{\ln2}\int_{0}^{\infty }\frac{P\left(SINR⩾t\right)}{1+t}dt. \end{array}$$ (16)

Thus, under GBS coverage, the traversal rate is:


$$\begin{array}{c} {ℛ}_{B}=\frac{E\left[\min\left(M,N_{B}\right)\right]}{M\ln2}\int_{0}^{\infty }\frac{P\left(SINR_{UE,B}⩾t\right)}{1+t}dt. \end{array}$$ (17)

Similarly, under UAV coverage, the traversal rate is:


$$\begin{array}{c} {𝒯}_{U}=\frac{E\left[\min\left(M,N_{U}\right)\right]}{M\ln2}\int_{0}^{\infty }\frac{P\left(SINR_{UE,U}⩾\gamma \right)}{1+t}dt. \end{array}$$ (18)

Therefore, for UAV-assisted coverage, the total network traversal rate is:


$$\begin{array}{c} {ℛ}_{UU}={ℛ}_{B}+{ℛ}_{U}. \end{array}$$ (19)

For comparison, without UAV assistance, the network traversal rate is:


$$\begin{array}{c} {ℛ}_{NU}=\frac{E\left[\min\left(M,N_{NU}\right)\right]}{M\ln2}\int_{0}^{\infty }\frac{P\left(SINR_{UE,NU}⩾\gamma \right)}{1+t}dt. \end{array}$$ (20)

Network Throughput and Traversal Rate are two key indicators for network performance analysis, with distinct attributes and applicability. The former is more consistent with practical scenarios, and their core differences and practicality are summarized as follows.

Network Throughput is more consistent with reality: It directly corresponds to end-to-end network performance, user experience, and engineering needs (e.g., your UAV-terrestrial base station collaborative coverage research). It fully incorporates practical factors like inter-cell interference and resource allocation, which determine the real service capability of the network.

Traversal Rate serves as a theoretical basis: It is used for theoretical performance boundary derivation and algorithm optimization benchmarks, but cannot reflect the actual network performance due to ignoring practical constraints.

### Performance analysis

After presenting the network model, we analyze the key factors affecting the network performance metrics of throughput and traversal rate. In particular, the interference probability and the SINR experienced by users associated with the GBS and the UAV are examined.

### Interference probability

The UAV reuses the spectrum resources of the GBS. The time-frequency resources at each base station are rapidly allocated to the mobile users it serves. This spectrum sharing causes mutual interference between the UAV and the GBS. Therefore, it is necessary to first analyze and quantify the interference between them.

Define the access capacity of the GBS and UAV, i.e., the maximum number of users *M* that can be served on a single resource block. If the number of users to be served by the GBS or UAV exceeds its access capacity, a random scheduling scheme is applied to select any *M* users for service. The transmitting power of the GBS and UAV is denoted by *P*_*B*_ and *P*_*U*_, respectively, and is evenly distributed among their associated users. For a given channel of the GBS or UAV, the transmitting power is *P*_*B*_/*M* and *P*_*U*_/*M*, respectively. Considering the randomness in allocation, we analyze network performance using a single resource block as the reference.

For users covered by the GBS, the interference comes from other GBSs outside the cell and environmental noise. If the UAV assigns the same frequency band to users within its coverage, GBS users will also experience interference from the UAV. Let *N*_*U*_ denote the number of users within the UAV coverage area. The number of channels required by the UAV is $\min(M,N_{U})$. In this case, there are a total of $C_{M}^{\min(M,N_{U})}$ possible channel allocation combinations, and for a specific channel, the number of combinations in which it is used is $C_{M-1}^{\min(M,N_{U})-1}$. Due to the randomness of UAV channel allocation, the probability that a GBS channel is simultaneously used by the UAV is:


$$\begin{array}{c} P_{UI}=\frac{C_{M-1}^{\min\left(M,N_{U}\right)-1}}{C_{M}^{\min\left(M,N_{U}\right)}}=\frac{\min\left(M,N_{U}\right)}{M}. \end{array}$$ (21)

The area covered by the UAV is $\omega (R_{C}^{2}-R_{B}^{2})$. Since users are distributed according to HPPP within the community, the distribution of the number of users covered by the UAV at a given time can be expressed as:


$$\begin{array}{c} P\left(\;N_{U}=i\right)\;=\;\exp\left(-\omega \left(R_{C}^{2}-R_{B}^{2}\right)\right)\frac{\omega^{i}{\left(R_{C}^{2}-R_{B}^{2}\right)}^{i}}{i\text{!}}. \end{array}$$ (22)

Thus, $E\left[\min\left(M,N_{U}\right)\right]$ can be obtained by:


$$\begin{array}{cc} & E\left[\min\left(M,N_{U}\right)\right]\\ = & \sum_{i=0}^{\infty }\min\left(M,i\right)P\left(N_{U}=i\right)\\ = & \sum_{i=0}^{M_{tot}}\min\left(M,i\right)P\left(N_{U}=i\right)\\ & +\sum_{i=M+1}^{\infty }\min\left(M,i\right)P\left(N_{U}=i\right)\\ = & \sum_{i=0}^{M}iP\left(N_{U}=i\right)+M\sum_{i=M+1}^{\infty }P\left(N_{U}=i\right)\\ \overset{\left(a\right)}{=} & \sum_{i=0}^{M}iP\left(N_{U}=i\right)+M\left(1-\sum_{i=0}^{M}P\left(N_{U}=i\right)\right)\\ = & M-\sum_{i=0}^{M}\left(M-i\right)P\left(N_{U}=i\right), \end{array}$$ (23)

where step (a) uses the probability distribution property $\sum_{i=0}^{\infty }P(N_{U}=i)=1$, allowing the calculation to be performed with a finite sum, avoiding an infinite summation. Therefore, in an average sense, the probability that a certain GBS channel is also used by UAVs is:


$$\begin{array}{c} P_{UI}\;=\;\frac{E\left[\min\left(M,N_{U}\right)\right]}{M}=\;1\;-\;\sum_{i=0}^{M}\frac{M-i}{M}P\left(N_{U}=i\right). \end{array}$$ (24)

Similarly, for the area covered by the GBS at the center of the cell, which is $\pi R_{B}^{2}$, the distribution of the number of users *N*_*B*_ in the coverage area is:


$$\begin{array}{c} P\left(N_{B}=i\right)=\exp\left(-\pi R_{B}^{2}\right)\frac{\pi^{i}R_{B}^{2i}}{i\text{!}}. \end{array}$$ (25)

Correspondingly, $E\left[\min\left(M,N_{B}\right)\right]$ can be calculated by:


$$\begin{array}{c} E\left[\min\left(M,N_{B}\right)\right]=M-\sum_{i=0}^{M}\left(M-i\right)P\left(N_{B}=i\right). \end{array}$$ (26)

For users covered by the UAV, the probability that a GBS channel in the center of the cell causes interference is:


$$\begin{array}{c} P_{BI}=1-\sum_{i=0}^{M}\frac{M-i}{M}P\left(N_{B}=i\right). \end{array}$$ (27)

If no UAV is used for auxiliary coverage, the GBS at the center of the cell covers an area with radius *R*_*C*_. The distribution of the number of users *N*_*NU*_ in this area is:


$$\begin{array}{c} P\left(N_{NU}=i\right)=\exp\left(-\pi R_{C}^{2}\right)\frac{\pi^{i}R_{C}^{2i}}{i\text{!}}. \end{array}$$ (28)

Thus, we can obtain:


$$\begin{array}{c} E\left[\min\left(M,N_{NU}\right)\right]=M-\sum_{i=0}^{M}\left(M-i\right)P\left(N_{NU}=i\right). \end{array}$$ (29)

### SINR analysis of GBS users

Considering the random distribution of users and the circular symmetry of the region, we first analyze the users whose distance from the GBS at the center of the cell is *R*. The Laplace transform of the interference from the UAV, $I_{UI}\left(R,s\right)$, can be expressed as:


ℒUI(R,s)=E[e−sIUI(R)]=(a)PUIE[e−sIUI(R)|IUI≠0]+(1−PUI)E[e−sIUI(R)|IUI=0]ℒUI(R,s)=PUIE[e−sIUI(R)|IUI≠0]+1−PUI, (30)

where step (*a*) is obtained by applying formula (24) and splitting *I*_*UI*_ into the cases of zero and non-zero interference.

For a given user, since the UAV moves along a circular trajectory, its position at any time can be modeled as a uniform distribution along the orbit. Let θ denote the angle between the UAV and the target user with respect to the coordinate origin, which is uniformly distributed in [0,2π). The horizontal distance between the UAV and the GBS user is then:


$$\begin{array}{c} Z_{U}(R,\theta )=\sqrt{R_{U}^{2}+R^{2}-2R_{U}R\cos\theta }. \end{array}$$ (31)

Further, when $I_{UI}\neq 0$, we have:


$$\begin{array}{cc} & E\left[e^{-sI_{UI}\left(R\right)}|I_{UI}\neq 0\right]\\ \overset{\left(a\right)}{=} & P_{U,L}\left(R\right)E_{I_{G,UI}}\left[e^{-sI_{UI}\left(R\right)}|I_{UI}\neq 0,Los\right]\\ & +P_{U,N}\left(R\right)E_{I_{UI}}\left[e^{-sI_{UI}\left(R\right)}|I_{UI}\neq 0,NLos\right], \end{array}$$ (32)

where step (a) is obtained by conditioning UAV interference on whether the interfering link is LoS or NLoS.

We now derive the expression for the LoS case. When the interfering UAV link is LoS, the corresponding conditional Laplace transform is given by:


=EIUI[e−sIUI(R)|IUI≠0,Los]=Eθ,gL[exp(−sηLPU(ZU(R,θ)2+H2)−αL/2gL)]=(a)12π∫02πPEgL[exp(−sηLPU(ZU(R,θ)2=+H2)−αL/2gL)]dθ=(b)12π∫02π∫0∞exp(−sηLPU(ZU(R,θ)2+H2)−αL/2x)=×mLmLxmL−1𝛤(mL)exp(−mLx)dxdθ=(c)1πmLmL𝛤(mL)∫0π∫0∞exp{[−mL−sηLPU(RU2+R2−2RURcosθ+H2)−αL/2]x}×xmL−1dxdθ=(d)1πmLmL𝛤(mL)∫0π[mL+sηLPU(RU2+R2−2RURcosθ=+H2)−αL/2]−mLdθ∫0∞tmL−1exp(−t)dt=mLmLπ∫0π[mL+sηLPU(RU2+R2−2RURcosθ+H2)−αL/2]−mLdθ, (33)

where step (a) is obtained according to the uniform distribution of the angle θ between the UAV and the user, step (b) follows by incorporating the small-scale fading model of the Nakagami-m distribution with parameter *m*_*L*_, step (c) arises from the symmetry of the included angles, and step (d) is obtained by applying the following substitution:


t=x[mL+SηLPU(RU2+R2−2RURcosθ=+H2)−αL/2]−mL. (34)

Similarly, for the NLoS case of UAV interference, we obtain:


=EIUI[e−sIUI(R)|IUI≠0,NLos]=mNmN2π∫02π[mN+sηNPU(RU2+R2−2RURcosθ=+H2)−αN/2]−mNdθ. (35)

By substituting the above expression into (32), the conditional expectation can be written as:


$$\begin{array}{cc} & E\left[e^{-sI_{UI}\left(R\right)}|I_{UI}\neq 0\right]\\ = & P_{U,L}\left(R\right)\frac{m_{L}^{m_{L}}}{\pi }\int_{0}^{\pi }[m_{L}+s\eta_{L}P_{U}(R_{U}^{2}+R^{2}\\ & {{-2R_{U}R\cos\theta +H^{2})}^{-\alpha_{L}/2}]}^{-m_{L}}d\theta \\ & +P_{U,N}\left(R\right)\frac{m_{N}^{m_{N}}}{\pi }\int_{0}^{\pi }[m_{N}+s\eta_{N}P_{U}(R_{U}^{2}+\\ & {{R^{2}-2R_{U}R\cos\theta +H^{2})}^{-\alpha_{N}/2}]}^{-m_{N}}d\theta . \end{array}$$ (36)

Thus, ${ℒ}_{UI}\left(R,s\right)$ can be transformed as:


=ℒUI(R,s)=PUIPU,L(R)mLmLπ∫0π[mL+sηLPU(RU2+R2=−2RURcosθ+H2)−αL/2]−mLdθ=+PUIPU,N(R)mNmNπ∫0π[mN+sηNPU(RU2+R2=−2RURcosθ+H2)−αN/2]−mNdθ+1−PUI. (37)

Consequently, when the distance between the user and the GBS at the cell center is *R*, the SINR distribution can be written as:


=P(SINRUE,B⩾γ|R)=P(PUE,B(R)IRC(R)+IUI(R)+N0⩾γ)=(a)PB,L(R)P(ηLPBR−αLgLIRC(R)+IUI(R)+N0⩾γ)==+PB,N(R)P(ηNPBR−αNgNIRC(R)+IUI(R)+N0⩾γ)=PB,L(R)P(gL⩾γRαLηLPB(IRC(R)+IUI(R)+N0))==+PB,N(R)P(gN⩾γRαNηNPB(IRC(R)+IUI(R)+N0)), (38)

where step (a)is derived by distinguishing whether the desired signal is received through a LoS or an NLoS channel.

For the LoS case, the coverage probability can be evaluated as follows:


=P(gL⩾γRαLηLPB(IRC(R)+IUI(R)+N0))==(a)∑n=1mLCnmL(−1)n+1exp(−nDmLγRαLηLPB(IRC(R)=+IUI(R)+N0IRC(R))nDmLγRαLηLPB)=(b)∑n=1mLCnmL(−1)n+1exp(−nDmLγRαLηLPBN0)=×ℒRC(R,nDmLγRαLηLPB)ℒUI(R,nDmLγRαLηLPB), (39)

where step (a)employs the CCDF approximation of a gamma-distributed fading variable with $D_{m}={\left(m!\right)}^{-\frac{1}{m}}m$, step (b) follows directly from the definition of the Laplace transform.

Let *P*_*G*_ denote the transmit power of external interfering GBSs. According to [28], the Laplace transform of the interference is given by:


ℒRC(R,s)=exp(∫0π∫1ZG−αN(R,θ)sPG+11t2αN−1(1−t)−2αNdtdθ−2λGαN(sPG)2αN×∫0π∫1ZG−αN(R,θ)sPG+11t2αN−1(1−t)−2αNdtdθ), (40)

where


$$\begin{array}{c} Z_{G}(R,\theta )=\sqrt{R_{C}^{2}-R^{2}\sin^{2}\theta }-R\cos\theta . \end{array}$$ (41)

Similarly, the NLoS coverage probability is given by:


=P(gN⩾γRαNηNPB(IRC(R)+IUI(R)+N0))==∑n=1mNCnmN(−1)n+1exp(−nDmNγRαNηNPBN0)=×ℒRC(R,nDmNγRαNηNPB)ℒUI(R,nDmNγRαNηNPB). (42)

Therefore, when a user is located at distance *R* from the central GBS, the SINR distribution is:


=P(SINRUE,B⩾γ|R)=exp(−βR)∑n=1mLCnmL(−1)n+1exp(−nDmLγRαLηLPBN0)=×ℒRC(R,nDmLγRαLηLPB)ℒUI(R,nDmLγRαLηLPB)=+(1−exp(−βR))∑n=1mNCnmN(−1)n+1=×exp(−nDmNγRαNηNPBN0)ℒRC(R,nDmNγRαNηNPB)×ℒUI(R,nDmNγRαNηNPB). (43)

According to the nature of HPPP, any user covered by a GBS is uniformly distributed within its coverage area. Therefore, the probability density function (PDF) of the distance *R* from the user to the base station is:


$$\begin{array}{c} f_{B,R}\left(r\right)=\frac{2r}{R_{B}^{2}}. \end{array}$$ (44)

Thus, the SINR distribution for an arbitrary user covered by the GBS can be expressed as:


=P(SINRUE,B⩾γ)=∫0RBfR(r)P(SINRUE,B⩾γ|r)dr=∫0RB2rRB2{exp(−βr)∑n=1mLCnmL(−1)n+1=×exp(−nDmLγrαLηLPBN0)ℒRC(r,nDmLγrαLηLPB)=×ℒUI(r,nDmLγrαLηLPB)=+(1−exp(−βr))=×∑n=1mNCnmN(−1)n+1exp(−nDmNγrαNηNPBN0)=×ℒRC(r,nDmNγrαNηNPB)ℒUI(r,nDmNγrαNηNPB)}dr. (45)

In contrast, when UAV assistance is not deployed, the central GBS randomly allocates channels to serve users across the entire cell. In this case, users are not affected by UAV co-channel interference. The corresponding SINR distribution is:


=P(SINRUE,NU⩾γ)=∫0RC2rRC2{exp(−βr)∑n=1mLCnmL(−1)n+1=×exp(−nDmLγrαLηLPBN0)ℒRC(r,nDmLγrαLηLPB)=+(1−exp(−βr))∑n=1mNCnmN(−1)n+1=×exp(−nDmNγrαNηNPBN0)ℒRC(r,nDmNγrαNηNPB)}dr. (46)

### SINR analysis of UAV users

Because the UAV moves around the center of the region at a constant angular velocity, and the users within the covered annular area are assigned channels uniformly at random, the user distribution is statistically symmetric with respect to the line connecting the UAV and the region center. Therefore, at any given time, the joint distributionof the user’s angle 𝛩 and radial distance *R* from the center is given by:


$$\begin{array}{c} F_{U,R,𝛩}\left(R⩽r,𝛩⩽\theta \right)=\frac{\left(\theta +\omega \right)\left(r^{2}-R_{B}^{2}\right)}{2\omega \left(R_{C}^{2}-R_{B}^{2}\right)}, \end{array}$$ (47)

where θ∈[−ω,ω] and $r\in \left[R_{B},R_{C}\right]$. From the joint cumulative distribution, the corresponding probability density function is derived as:


$$\begin{array}{c} f_{U,R,𝛩}\left(r,\theta \right)=\frac{r}{\omega \left(R_{C}^{2}-R_{B}^{2}\right)}. \end{array}$$ (48)

By integrating this joint density over 𝛩 and *r*, respectively, the marginal probability density functions are obtained as:


$$\begin{array}{c} f_{U,R}\left(r\right)=\frac{2r}{\left(R_{C}^{2}-R_{B}^{2}\right)}, \end{array}$$ (49)

and


$$\begin{array}{c} f_{U,𝛩}\left(r\right)=\frac{1}{2\omega }. \end{array}$$ (50)

Thus, the joint density can be expressed as


$$\begin{array}{c} f_{U,R,𝛩}\left(r,\theta \right)=f_{U,R}\left(r\right)f_{U,𝛩}\left(\theta \right), \end{array}$$ (51)

which demonstrates that the random variables 𝛩 and *R* are statistically independent.

The Laplace transform of the interference generated by the drone to a user located at distance *R*, denoted as *I*_*U*,*BI*_, is given by:


ℒBI(R,s)=E[e−sIBI(R)]=(a)PBIE[e−sIBI(R)|IBI≠0]=+(1−PBI)E[e−sIUI(R)|IU,BI=0]=PBIE[e−sIBI(R)|IUI≠0]+1−PBI, (52)

where step (a) is obtained according to whether *I*_*BI*_ equals zero and based on (27). When $I_{BI}\neq 0$, the contribution from the GBS is separated into LoS and NLoS components, yielding:


=E[e−sIBI(R)|IBI≠0]=PB,L(R)EIU,BI[e−sIBI(R)|IBI≠0,Los]+PB,N(R)EIU,BI[e−sIBI(R)|IBI≠0,NLos] (53)

We first derive the LoS interference term. Because the small-scale fading follows a Nakagami-*m* distribution with parameter *m*_*L*_, the expectation becomes


=EIBI[e−sIBI(R)|IBI≠0,Los]=EgL[exp(−sηLPBR−αLgL)]=∫0∞exp(−sηLPBR−αLx)mLmLxmL−1𝛤(mL)exp(−mLx)dx=mLmL𝛤(mL)∫0∞exp[(−mL−sηLPBR−αL)x]xmL−1dx=mLmL𝛤(mL)(mL+sηLPBR−αL)−mL∫0∞tmL−1exp(−t)dt=(mLmL+sηLPBR−αL)mL, (54)

where


$$\begin{array}{c} t=x\left(m_{L}+s\eta_{L}P_{U}R^{-\alpha_{L}}\right). \end{array}$$ (55)

Similarly, the Laplace transform of the interference from the GBS under the NLoS condition is given by:


$$\begin{array}{cc} & E_{I_{BI}}\left[e^{-sI_{BI}\left(R\right)}|I_{BI}\neq 0,NLos\right]\\ = & {\left(\frac{m_{N}}{m_{N}+s\eta_{N}P_{B}R^{-\alpha_{L}}}\right)}^{m_{N}}, \end{array}$$ (56)

which is obtained following the same derivation steps as in the LoS case, noting that the small-scale fading follows a Nakagami-*m* distribution with parameter *m*_*N*_ under the NLoS condition.

By substituting (53), the conditional Laplace transform for the non-zero interference case is written as:


$$\begin{array}{cc} & E\left[e^{-sI_{BI}\left(R\right)}|I_{BI}\neq 0\right]\\ \overset{\left(a\right)}{=} & P_{B,L}\left(R\right){\left(\frac{m_{L}}{m_{L}+s\eta_{L}P_{B}R^{-\alpha_{L}}}\right)}^{m_{L}}\\ & +P_{B,N}\left(R\right){\left(\frac{m_{N}}{m_{N}+s\eta_{N}P_{B}R^{-\alpha_{L}}}\right)}^{m_{N}}. \end{array}$$ (57)

Thus, the Laplace transform of the interference from the GBS is:


ℒBI(R,s)=PBIPB,L(R)(mLmL+sηLPBR−αL)mL=+PBIPB,N(R)(mNmN+sηNPBR−αL)mN+1−PBI, (58)

which yields the final closed-form shown above.

The distribution of the UAV-user SINR for a fixed radial distance *R* and an angle 𝛩 can be expressed as:


P(SINRUE,U⩾γ|R,𝛩)=P(PUE,U(R,𝛩)IRC(R,𝛩)+IBI(R,𝛩)+N0⩾γ)=(a)P(PUE,U(R,𝛩)IRC(R)+IBI(R)+N0⩾γ)=(b)PU,L(ZU(R,𝛩))×P(ηLPB(ZU2(R,𝛩)+H2)−αL/2gLIRC(R)+IBI(R)+N0⩾γ)=+PU,N(ZU(R,𝛩))×P(ηNPB(ZU2(R,𝛩)+H2)−αN/2gNIRC(R)+IBI(R)+N0⩾γ)=PU,L(ZU(R,𝛩))P(gL⩾γ(ZU2(R,𝛩)+H2)αL/2ηLPB=(IRC(R)+IBI(R)+N0)gL⩾γ(ZU2(R,𝛩)+H2)αL/2ηLPB)+PU,N(ZU(R,𝛩))=×P(gN⩾γ(ZU2(R,𝛩)+H2)αN/2ηNPB×(IRC(R)+IBI(R)+N0)gL⩾γ(ZU2(R,𝛩)+H2)αL/2ηLPB), (59)

where step (*a*) uses the fact that the interference from out-of-cell base stations, $I_{R_{C}}$, and from the cell-center base station, *I*_*BI*_, is statistically independent of the included angle 𝛩, and therefore only depends on the horizontal distance *R*. Step (*b*) separates the received signal into the LoS and NLoS cases, and evaluates the corresponding conditional SINR distributions individually.

For the LoS case, the coverage probability can be computed as:


P(gL⩾γ(ZU2(R,𝛩)+H2)αL/2ηLPB×(IRC(R)+IBI(R)+N0)γ(ZU2(R,𝛩)+H2)αL/2ηLPB)=(a)∑n=1mLCnmL(−1)n+1exp(−nDmLγ(ZU2(R,𝛩)+H2)αL/2ηLPB=(IRC(R)+IBI(R)+N0)nDmLγ(ZU2(R,𝛩)+H2)αL/2ηLPB)=(b)∑n=1mLCnmL(−1)n+1=×exp(−nDmLγ(ZU2(R,𝛩)+H2)αL/2ηLPBN0)=×ℒRC(R,nDmLγ(ZU2(R,𝛩)+H2)αL/2ηLPB)=×ℒBI(R,nDmLγ(ZU2(R,𝛩)+H2)αL/2ηLPB), (60)

where step (a) uses the approximate formula $D_{m}=(m!{)}^{-\frac{1}{m}}m$ for the CCDF of the gamma distribution, while step (b) is expressed in terms of the Laplace transform.

Similarly, for the NLoS case, we have:


P(gN⩾γ(ZU2(R,𝛩)+H2)αN/2ηNPB×(IRC(R)+IBI(R)+N0)gN⩾γ(ZU2(R,𝛩)+H2)αN/2ηNPB)=∑n=1mNCnmN(−1)n+1=×exp(−nDmNγ(ZU2(R,𝛩)+H2)αN/2ηNPBN0)=×ℒRC(nDmNγ(ZU2(R,𝛩)+H2)αN/2ηNPB)=×ℒBI(R,nDmNγ(ZU2(R,𝛩)+H2)αN/2ηNPB). (61)

Therefore, when the UAV-user distance is *R* and the included angle is 𝛩, the SINR distribution can be written as:


P(SINRUE,U⩾γ|R,𝛩)=11+cexp(−b(φ(HZU(R,𝛩))−c))∑n=1mLCnmL(−1)n+1=×exp(−nDmLγ(ZU2(R,𝛩)+H2)αL/2ηLPBN0)=×ℒRC(R,nDmLγ(ZU2(R,𝛩)+H2)αL/2ηLPB)=×ℒBI(R,nDmLγ(ZU2(R,𝛩)+H2)αL/2ηLPB)=+(1−11+cexp(−b(φ(HZU(R,𝛩))−c)))×∑n=1mNCnmN(−1)n+1×exp(−nDmNγ(ZU2(R,𝛩)+H2)αN/2ηNPBN0)×ℒRC(nDmNγ(ZU2(R,𝛩)+H2)αN/2ηNPB)×ℒBI(R,nDmNγ(ZU2(R,𝛩)+H2)αN/2ηNPB). (62)

Finally, the SINR distribution for any UAV-covered user can be obtained by:


$$\begin{array}{cc} & P\left(SINR_{UE,U}⩾\gamma \right)=\int_{-\omega }^{+\omega }\int_{R_{B}}^{R_{C}}f_{R,𝛩}\left(r,\theta \right)\\ & \times P\left(SINR_{UE,U}⩾\gamma |r,\theta \right)drd\theta . \end{array}$$ (63)

### Simulation results

**Fig 2:**
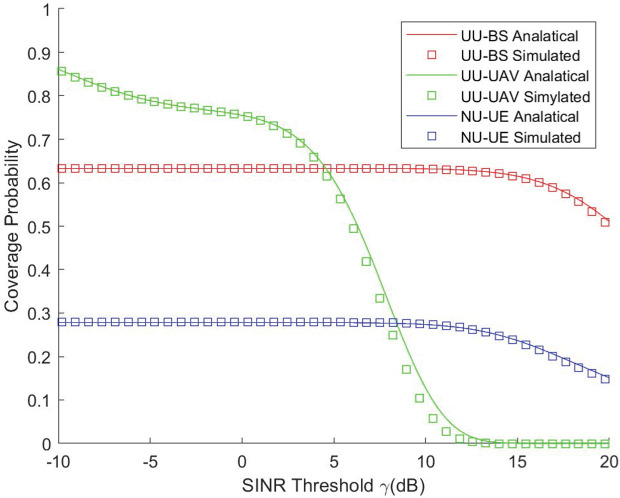
Coverage probability vs. SINR threshold.

**Fig 3:**
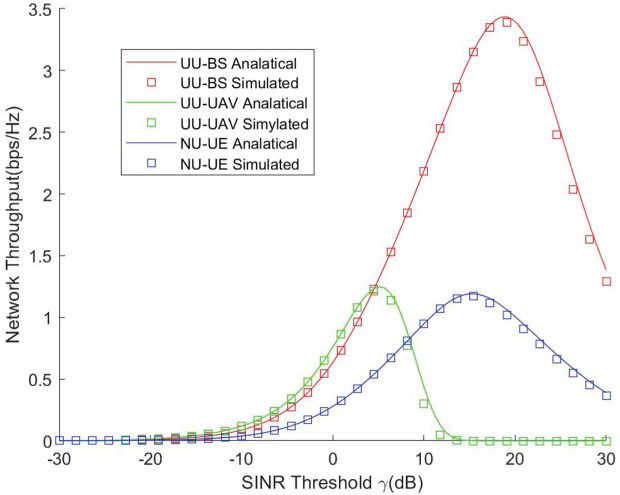
Network throughput vs. SINR threshold.

**Fig 4:**
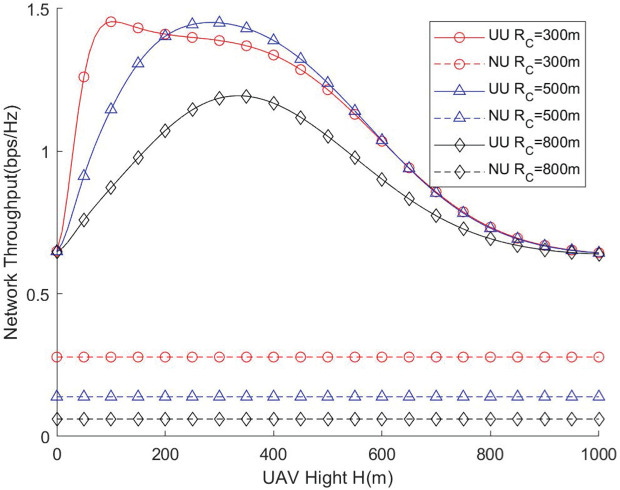
Network throughput vs. UAV height.

**Fig 5:**
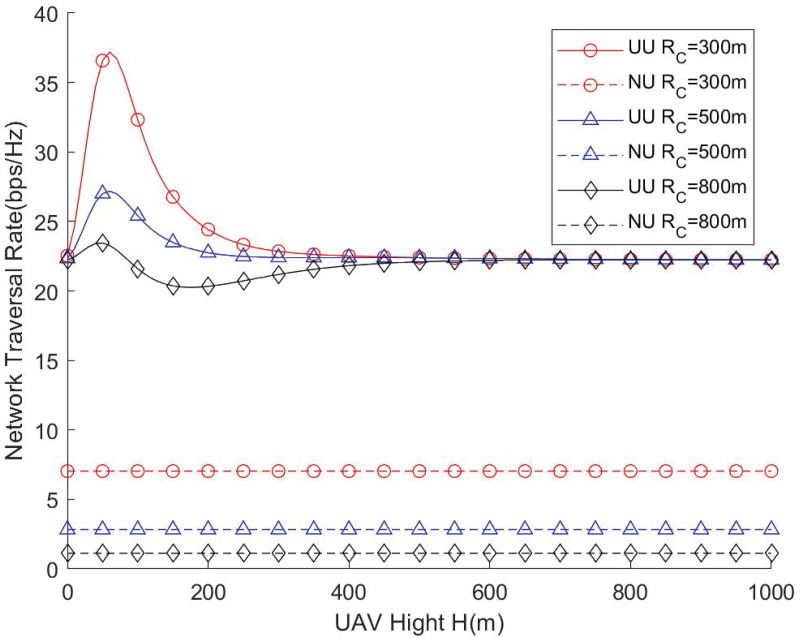
Network traversal rate vs. UAV height.

**Fig 6:**
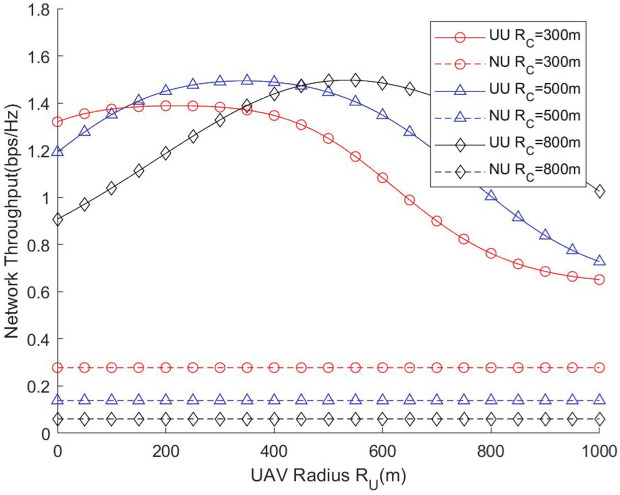
Network throughput vs. UAV radius.

**Fig 7:**
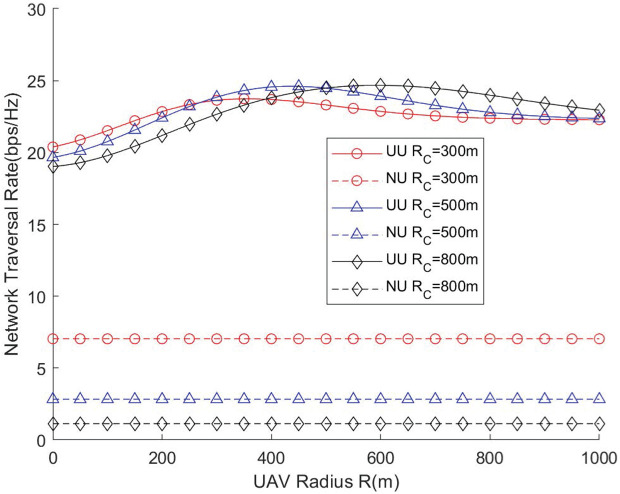
Network traversal rate vs. UAV radius.

**Fig 8:**
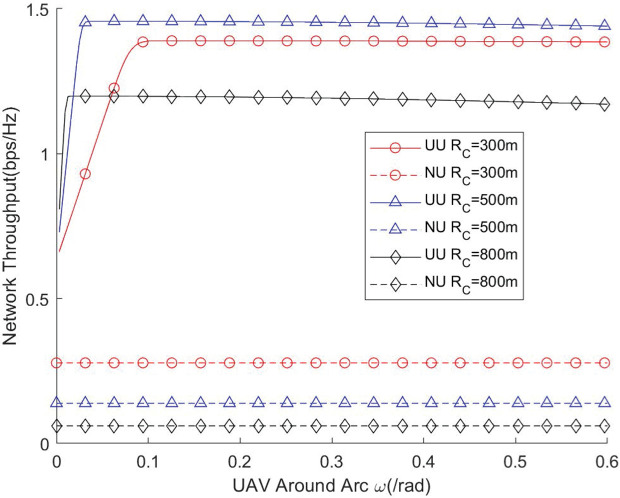
Network throughput vs. UAV around arc.

**Fig 9:**
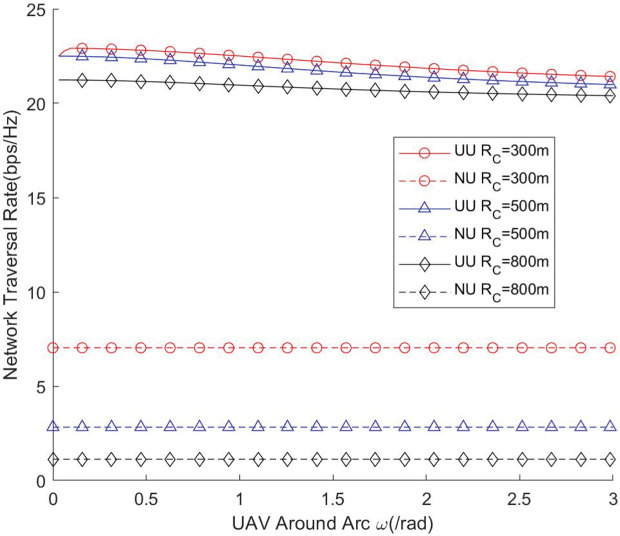
Network traversal rate vs. UAV around arc.

**Fig 10:**
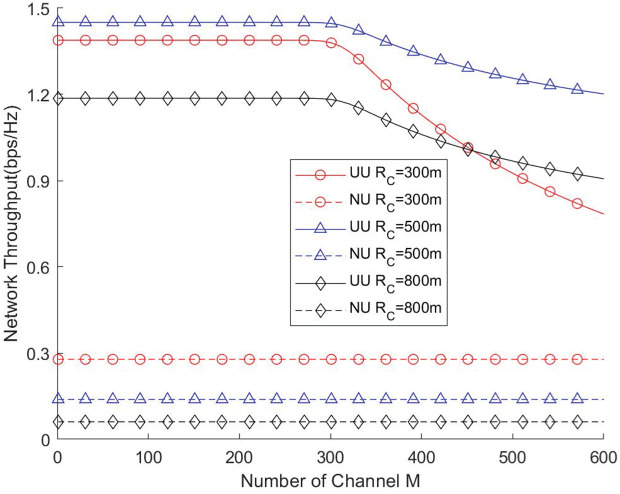
Network throughput vs. Number of channel.

**Fig 11:**
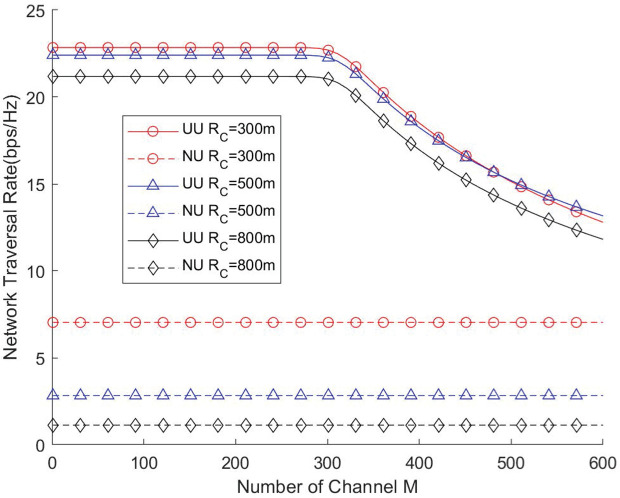
Network traversal rate vs. Number of channel.

**Fig 12:**
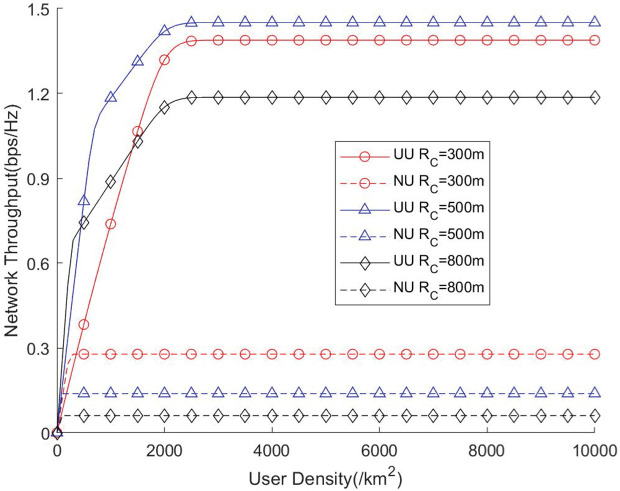
Network throughput vs. User density.

**Fig 13:**
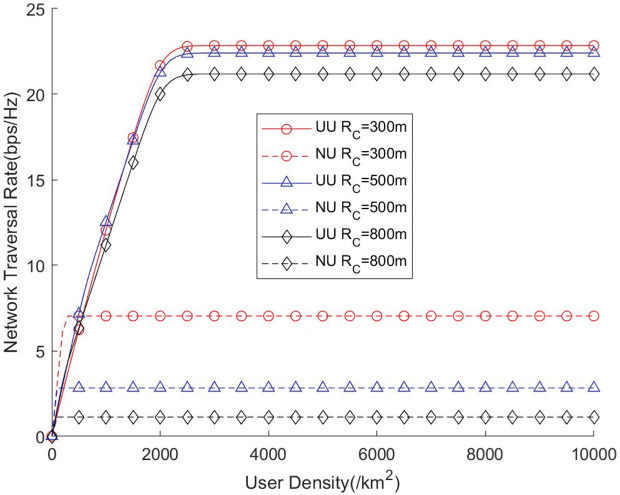
Network traversal rate vs. User density.

In this section, we first introduce the simulation method for the UAV-assisted edge coverage scenario. Based on this setup, the theoretical derivations presented above are verified through simulation. After confirming the accuracy of the theoretical analysis, the impact of various network parameters, such as including UAV flight altitude, UAV hovering radius, coverage radius of the GBS at the cell center, and user density, on network performance is evaluated using the theoretical results. For each numerical scenario, a baseline case without UAV assistance is also provided for comparison. The results demonstrate that UAV-assisted coverage can significantly enhance the network’s coverage performance. Unless otherwise specified, the parameter settings used in the analysis of individual factors are summarized in Table 1 [10]. Besides, the source code for the simulation results (i.e., Figs 2–13) are provided in the S1 File.

**Table 1:** Simulation Settings.

variable	value	variable	value
*b*	0.136	$\lambda_{UE}$	10000/*km*^2^
*c*	11.95	$\lambda_{G}$	25/*km*^2^
1/β	3141.4	*P*_*B*_	20 *W*
$\alpha_{L}$	2.5	*P*_*U*_	5 *W*
$\alpha_{N}$	4	*P*_*G*_	20 *W*
$\eta_{L}$	10^−5.11^	*N*_0_	−84*dBm*
$\eta_{N}$	10^−10^	*H*	300 *m*
*m*_*L*_	3	*R*_*B*_	100 *m*
*m*_*N*_	1	*R*_*B*_	200 *m*
*γ*	1	*R*_*B*_	300 *m*
*ω*	π/8	*M*	64

First of all, it is necessary to establish a simulation scenario. By specifying parameters such as the cell radius, the coverage radius of the GBS after partitioning, the UAV flight altitude, and its hovering radius, the coverage areas of both the GBS and the UAV can be determined. To model the widespread interference from GBSs outside the cell, the entire simulation area is defined as a square with a side length of 20,000 m. In this case, the spatial distribution of the interfering GBSs is irregular. To facilitate simulation, the same method introduced in the the Performance Analysis section is used to generate the locations of these interfering GBSs.

Within the cell, the number of users is first generated according to a Poisson distribution, based on the user density and the cell area. The generated users are then uniformly distributed throughout the cell. Given the network parameters, the number and positions of users covered by the GBS and the UAV located at the cell center can be determined.

Based on the total number of available channels, the allocation of users and channels for both the GBS and the UAV is performed. For users under GBS coverage, potential interference from the UAV is determined according to the assigned channel. The SINR of each user is then calculated to evaluate whether the user is successfully covered according to a predefined SINR threshold. In each iteration, the numbers of users who acquire channels and those who are successfully covered are recorded separately.

The above process is repeated for 10,000 iterations. By dividing the total number of users that acquire channels by the total number of successfully covered users, the successful coverage probability for any user under GBS coverage can be obtained. Furthermore, the aggregate throughput under GBS coverage is computed by multiplying the total number of successfully covered users by the corresponding communication rate and channel bandwidth, then dividing by the number of simulation iterations. An analogous procedure is applied to evaluate the UAV-assisted coverage scenario.

Fig 2 illustrates both the simulation and theoretical results for the coverage provided by the UAV and the GBS under different SINR thresholds, as well as the coverage performance of a single GBS without UAV assistance. It shows the performance of the UU-UAV is better than others with low SINR threshold and the condition is reverse with high SINR threshold. This is because UAV communications are more sensitive to the SINR threshold. UAVs fly at high altitudes and are in a constant state of motion. When the SINR threshold is low, the advantages of its LoS channel dominate, thus enabling superior performance. However, as the SINR increases, the bit error rate rises rapidly due to its mobility characteristics, leading to a sharp decline in performance. These features result in that the performance of the UU-UAV is better than others with low SINR threshold and the condition is reverse with high SINR threshold.

Fig 3 presents the simulation and theoretical results of the network throughput as a function of the SINR threshold for the UAV and the GBS in the UAV-assisted network, alongside the throughput performance of the GBS when the UAV is not deployed. It can be observed that the theoretical analysis closely matches the simulation results, validating the accuracy of the derived models. From the coverage results, UAV deployment significantly improves user coverage. However, the UAV coverage degrades more rapidly as the SINR threshold increases. For GBSs, coverage performance also benefits from UAV assistance because UAVs can provide coverage to users in areas that would otherwise be limited to smaller regions by the GBS alone. Regarding network throughput, the UAV’s performance is more sensitive to the SINR threshold, resulting in a lower maximum throughput compared with GBSs. Nevertheless, at lower threshold values, UAV-assisted coverage can achieve throughput comparable to that of a GBS operating without UAV assistance, demonstrating the effectiveness of UAV deployment in enhancing network performance under stringent coverage requirements.

Fig 4 and Fig 5 illustrate the relationship between the UAV flight height and the network throughput as well as the network coverage ratio under different cell radii. From the results, it is evident that there exists an optimal UAV altitude that maximizes both coverage and overall network performance. The assistance of UAVs provides a significant enhancement in network performance compared with scenarios without UAV deployment, demonstrating the effectiveness of UAV-assisted coverage strategies. As the cell radius increases, the UAV needs to operate at a higher altitude to maintain reliable communication links across a wider service area, ensuring that users at the cell edge receive sufficient signal strength. Conversely, if the UAV flight height is excessively large, the distance-dependent path loss and reduced received signal power may limit coverage efficiency, and the effect of the cell radius on network performance becomes negligible. Therefore, careful optimization of UAV altitude is crucial during deployment to achieve a balance between coverage area, signal quality, and overall throughput. This highlights that UAV deployment strategies should consider both the geometric parameters of the network and the propagation characteristics to fully leverage UAV mobility for performance enhancement.

Fig 6 and Fig 7 illustrate how the UAV’s circular flight (surround) radius affects the network throughput and traversal rate under different cell radii. First, it can be observed from Fig 6 that for a given coverage radius of GBS, there exists an optimal flight radius for the UAV that maximizes the network throughput. This is because an excessively small UAV flight radius will lead to severe interference between the GBS and the UAV, and the UAV will fail to provide adequate coverage for cell-edge users, resulting in a low network throughput. Conversely, an excessively large UAV flight radius will cause intense inter-cell interference with adjacent GBSs, which will also reduce the network throughput. In addition, it can be found that the larger the coverage radius of the GBS is, the larger the optimal flight radius of the UAV becomes accordingly, which indicates that there exists a set of optimal parameter configurations between the two. These results indicate that the surround radius must be carefully adapted to the cell size, otherwise the UAV may fail to effectively assist users at the coverage boundary. This highlights the necessity of designing adaptive UAV deployment strategies for networks with varying spatial scales.

Fig 8 and Fig 9 illustrate how the angular span (radian) of the UAV’s instantaneous coverage region impacts network throughput and traversal rate under different cell radii. The results demonstrate that the UAV’s coverage radian must be carefully configured to fully exploit the available network resources. If the coverage radian is set too small, the number of users falling within the instantaneous coverage region becomes insufficient, resulting in underutilization of channel resources and consequently lower throughput. Conversely, when the coverage radian is excessively large, the UAV must serve a larger set of users simultaneously. In this case, many users may lie far from the UAV, leading to degraded link quality and reduced coverage probability. This distance-induced attenuation ultimately diminishes the achievable network performance. These observations indicate that there exists an optimal coverage radian that balances user density and link quality. Properly determining this radian is therefore essential for maximizing UAV-assisted network efficiency, particularly in scenarios with different cell sizes.

Fig 10 and Fig 11 depict the impact of the available channel count on network throughput and traversal rate under different cell radii. The results indicate that the number of channels must be carefully configured to balance resource utilization and coverage performance. When the channel count is relatively small, increasing the number of channels does not significantly degrade network performance. This is because, under high user density, all channels remain fully occupied and operate at maximum load. However, when the number of channels becomes excessively large, a substantial portion of them remains idle due to insufficient instantaneous user demand, leading to underutilization of network resources. Overall, these findings show that an optimal channel configuration should be determined based on cell radius and user density to maximize the efficiency of UAV-assisted networks.

Fig 12 and Fig 13 illustrate the influence of varying user densities on network throughput and traversal rate. As the user density increases, the communication resources of the network become progressively more utilized, leading to a gradual improvement in the effective use of available channels. However, when the user density becomes excessively high, the network enters a full-load operating state, where all channels are continuously occupied. In this regime, users must compete for limited channel resources, which may degrade the per-user coverage probability and transmission quality. To enhance the network’s coverage capability under such high-load conditions, increasing the number of available channels becomes an effective strategy, as it enables the network to serve more users simultaneously and alleviates resource contention. These results highlight the importance of balancing user density and channel availability, suggesting that an appropriate scaling of channel resources is essential to maintaining stable coverage performance in UAV-assisted communication networks.

## Conclusion

This paper analyzed a heterogeneous network in which a UAV is employed to enhance cell-edge coverage. Using stochastic-geometry-based modeling, we evaluated the user coverage probability, network throughput, and traversal rate, and verified the analytical expressions through Monte Carlo simulations. The close match between analytical and simulation results confirms the accuracy of the proposed theoretical framework. The results show that UAV-assisted surround coverage effectively improves network performance. By reusing spectrum resources, the UAV extends coverage to additional users and divides the original cell into two functional regions: a central area served by the terrestrial base station and a ring-shaped area served by the UAV. This enables the terrestrial base station to allocate spectrum to users located at shorter distances, while the UAV provides reliable communication links for users distributed in the outer ring. The analysis further indicates that appropriate configuration of network parameters is essential for maximizing the performance gains of UAV-assisted coverage.

The future works can be explored as follows. First, this paper idealizes the target cell as a circular area. However, the target area is often irregular in actual network coverage. In such cases, due to the asymmetry of the area, UAV coverage from a central position or along a circular trajectory is not the optimal choice. A further extension of this work is to optimize the UAV’s movement trajectory for different coverage areas. On the other hand, this paper adopts the homogeneous Poisson point process (HPPP) model for modeling randomly distributed UAVs and terrestrial base stations, yet the actual deployment of UAVs or terrestrial base stations is not completely random. For example, considering mutual interference, the distance between individual base stations is often constrained. In this scenario, it is necessary to adopt the homogeneous hard-core point process (HCPP) for modeling, which ensures that the distance between any two base stations is greater than a specific threshold.

## Supporting information

S1 FileWe conduct simulations with MATLAB R2024b.The source code for Figs. 2–13 are provided in the supporting file S1.(PDF)

## References

[1] Harounabadi M, Heyn T. Toward Integration of 6G-NTN to Terrestrial Mobile Networks: Research and Standardization Aspects. IEEE Wireless Commun. 2023;30(6):20–6. doi: 10.1109/mwc.005.2300207

[2] Uwaechia AN, Mahyuddin NM. A Comprehensive Survey on Millimeter Wave Communications for Fifth-Generation Wireless Networks: Feasibility and Challenges. IEEE Access. 2020;8:62367–414. doi: 10.1109/access.2020.2984204

[3] Kaleem Z, Khalid W, Muqaibel A, Nasir AA, Yuen C, Karagiannidis GK. Learning-Aided UAV 3D Placement and Power Allocation for Sum-Capacity Enhancement Under Varying Altitudes. IEEE Commun Lett. 2022;26(7):1633–7. doi: 10.1109/lcomm.2022.3172171

[4] Ullah A, Choi W, Abbas ZH, Abbas G. Aerial-terrestrial networks with multi-antenna transmissions: how many UAVs need to be deployed?. IEEE Transactions on Vehicular Technology. 2023;73(2):2212–26.

[5] Huang H, Huang W. Cooperative selection and communications in 6G ultra-mmtc iov networks. In: 2024 International Symposium on Electrical, Electronics and Information Engineering (ISEEIE), 2024. 257–61.

[6] Dong W-Y, Yang S, Zhang P, Chen S. Stochastic Geometry Based Modeling and Analysis of Uplink Cooperative Satellite-Aerial-Terrestrial Networks for Nomadic Communications With Weak Satellite Coverage. IEEE J Select Areas Commun. 2024;42(12):3428–44. doi: 10.1109/jsac.2024.3459268

[7] Liu L, Zhang S, Zhang R. CoMP in the Sky: UAV Placement and Movement Optimization for Multi-User Communications. IEEE Trans Commun. 2019;67(8):5645–58. doi: 10.1109/tcomm.2019.2907944

[8] Jin W, Du C, Wang J, Wang S, Pan G, Niyato D. Multi-UAV CoMP Transmission Based on UAV Jitter Characteristics: Analysis and Optimization. IEEE Transactions on Wireless Communications. 2026;25:978–993.

[9] Sun H, Nan Y, Li Y, Wang X, Li S, Quek TQS. Uplink CoMP Transmission for Cellular-Connected UAV Networks. IEEE Wireless Commun Lett. 2023;12(9):1513–7. doi: 10.1109/lwc.2023.3281198

[10] Lyu J, Zeng Y, Zhang R. UAV-Aided Offloading for Cellular Hotspot. IEEE Trans Wireless Commun. 2018;17(6):3988–4001. doi: 10.1109/twc.2018.2818734

[11] Al-Hourani A, Kandeepan S, Lardner S. Optimal LAP Altitude for Maximum Coverage. IEEE Wireless Commun Lett. 2014;3(6):569–72. doi: 10.1109/lwc.2014.2342736

[12] Turgut E, Gursoy MC. Downlink Analysis in Unmanned Aerial Vehicle (UAV) Assisted Cellular Networks With Clustered Users. IEEE Access. 2018;6:36313–24. doi: 10.1109/access.2018.2841655

[13] Matracia M, Kishk MA, Alouini M-S. Coverage Analysis for UAV-Assisted Cellular Networks in Rural Areas. IEEE Open J Veh Technol. 2021;2:194–206. doi: 10.1109/ojvt.2021.3076814

[14] Zhang C, Zhang W. Spectrum Sharing for Drone Networks. IEEE J Select Areas Commun. 2016;:1–1. doi: 10.1109/jsac.2016.2633040

[15] Kim D, Lee J, Quek TQS. Multi-layer Unmanned Aerial Vehicle Networks: Modeling and Performance Analysis. IEEE Trans Wireless Commun. 2020;19(1):325–39. doi: 10.1109/twc.2019.2944378

[16] Chetlur VV, Dhillon HS. Downlink Coverage Analysis for a Finite 3D Wireless Network of Unmanned Aerial Vehicles. IEEE Trans Commun. 2017;:1–1. doi: 10.1109/tcomm.2017.2722500

[17] Zeng Y, Wu Q, Zhang R. Accessing From the Sky: A Tutorial on UAV Communications for 5G and Beyond. Proc IEEE. 2019;107(12):2327–75. doi: 10.1109/jproc.2019.2952892

[18] Matracia M, Kishk MA, Alouini M-S. UAV-Aided Post-Disaster Cellular Networks: A Novel Stochastic Geometry Approach. IEEE Trans Veh Technol. 2023;72(7):9406–18. doi: 10.1109/tvt.2023.3247920

[19] Armeniakos CK, Kanatas AG. Performance Comparison of Wireless Aerial 3D Cellular Network Models. IEEE Commun Lett. 2022;26(8):1779–83. doi: 10.1109/lcomm.2022.3181332

[20] Kouzayha N, ElSawy H, Dahrouj H, Alshaikh K, Al-Naffouri TY, Alouini M-S. Stochastic Geometry Analysis of Hybrid Aerial Terrestrial Networks with mmWave Backhauling. In: ICC 2020 - 2020 IEEE International Conference on Communications (ICC), 2020. 1–7. 10.1109/icc40277.2020.9148840

[21] Guo X, Zhang C, Yu F, Chen H. Coverage Analysis for UAV-Assisted mmWave Cellular Networks Using Poisson Hole Process. IEEE Trans Veh Technol. 2022;71(3):3171–86. doi: 10.1109/tvt.2021.3139776

[22] Ge X, Du B, Li Q, Michalopoulos DS. Energy Efficiency of Multiuser Multiantenna Random Cellular Networks With Minimum Distance Constraints. IEEE Trans Veh Technol. 2017;66(2):1696–708. doi: 10.1109/tvt.2016.2557359

[23] Deng N, Chen L, Wei H. A 3D UAV-assisted cellular network model with Inter-tier dependence. 2021 IEEE Wireless Communications and Networking, 2021.Conference (WCNC). 2021. 1–6.

[24] Junruo L, Yuanjie W, Qimei C, Yanzhao H, Xiaofeng T. Modeling and performance analysis of UAV-aided millimeter wave cellular networks with stochastic geometry. China Commun. 2024;21(6):146–62. doi: 10.23919/jcc.fa.2023-0290.202406

[25] Andrews JG, Bai T, Kulkarni M, Alkhateeb A, Gupta A, Heath RW. Modeling and Analyzing Millimeter Wave Cellular Systems. IEEE Trans Commun. 2016;:1–1. doi: 10.1109/tcomm.2016.2618794

[26] Khawaja W, Guvenc I, Matolak DW, Fiebig U-C, Schneckenburger N. A Survey of Air-to-Ground Propagation Channel Modeling for Unmanned Aerial Vehicles. IEEE Commun Surv Tutorials. 2019;21(3):2361–91. doi: 10.1109/comst.2019.2915069

[27] Bai T, Heath RW. Coverage and Rate Analysis for Millimeter-Wave Cellular Networks. IEEE Trans Wireless Commun. 2015;14(2):1100–14. doi: 10.1109/twc.2014.2364267

[28] Wang X, Zhang H, Leung VCM. Modeling and Performance Analysis of UAV-Assisted Cellular Networks in Isolated Regions. In: 2018 IEEE International Conference on Communications Workshops (ICC Workshops), 2018. 1–6. 10.1109/iccw.2018.8403636

