# Supplementary material for: Performance analysis of UAV-assisted coverage for cell edge
Source: PLoS One. 2026 May 14;21(5):e0346901. doi: 10.1371/journal.pone.0346901 (PMC13175502; doi:10.1371/journal.pone.0346901)
Supplement: S1 File — The source code for Figs. 2–13 are provided in the supporting file S1. (PDF) [file pone.0346901.s001.pdf]

- **Fig. 2: Coverage Probability vs. SINR threshold**

```
BS_I_lam=25e-6;
UE_lam=10000e-6;
KK_l=10.^(3./10);
KK_nl=10.^(23./10);
m_l=3;
m_nl=1;
a_l=2.5;
a_nl=4;
K_l=1./(KK_l.*(4.*pi.*2.*10.^9./(3.*10.^8)).^a_l);
K_nl=1./(KK_nl.*(4.*pi.*2.*10.^9./(3.*10.^8)).^a_nl);
P_UAV_UE=5;
H_UAV=300;
m_I=m_nl;
a_I=a_nl;
K_I=K_nl;
R_BS=100;
R_UAV=200;
R_0=300;
P_BS_UE=20;
P_BS_I=P_BS_UE;
N_cha=64;
UAV_cover_thet=pi./8;
sig2=10.^(-114./10);
gam_db=linspace(-30,30,500);
% gam_db=0;
UE_gam=10.^(gam_db./10);
% UE_gam=linspace(0,100,10)

[P_BS_a] =
R_BS_UE_ana(R_0,R_BS,R_UAV,H_UAV,UAV_cover_thet,N_cha,...
                           UE_lam,BS_I_lam,...
                           P_BS_UE,P_BS_I,P_UAV_UE,...
                           sig2,UE_gam,...

m_l,m_nl,a_l,a_nl,K_l,K_nl,...
                                       m_I,a_I,K_I);


[P_BS_s] =
R_BS_UE_simu(R_0,R_BS,R_UAV,H_UAV,UAV_cover_thet,N_cha,..
.
                           UE_lam,BS_I_lam,...
                           P_BS_UE,P_BS_I,P_UAV_UE,...
```

```
                                sig2,UE_gam,...

m_l,m_nl,a_l,a_nl,K_l,K_nl,...
                                               m_I,a_I,K_I);

[P_UAV_a] =
R_UAV_UE_ana(R_0,R_BS,R_UAV,H_UAV,UAV_cover_thet,N_cha,..
.
                                UE_lam,BS_I_lam,...
                                P_BS_UE,P_BS_I,P_UAV_UE,...
                                sig2,UE_gam,...

m_l,m_nl,a_l,a_nl,K_l,K_nl,...
                                               m_I,a_I,K_I);

[P_UAV_s] =
R_UAV_UE_simu(R_0,R_BS,R_UAV,H_UAV,UAV_cover_thet,N_cha,..
.
                                UE_lam,BS_I_lam,...
                                P_BS_UE,P_BS_I,P_UAV_UE,...
                                sig2,UE_gam,...

m_l,m_nl,a_l,a_nl,K_l,K_nl,...
                                               m_I,a_I,K_I);

[P_NU_a] =
NU_R_BS_UE_ana(R_0,R_BS,R_UAV,H_UAV,UAV_cover_thet,N_cha,.
..
                                UE_lam,BS_I_lam,...
                                P_BS_UE,P_BS_I,P_UAV_UE,...
                                sig2,UE_gam,...

m_l,m_nl,a_l,a_nl,K_l,K_nl,...
                                               m_I,a_I,K_I);

[P_NU_s] =
NU_R_BS_UE_simu(R_0,R_BS,R_UAV,H_UAV,UAV_cover_thet,N_cha,
...
                                UE_lam,BS_I_lam,...
                                P_BS_UE,P_BS_I,P_UAV_UE,...
                                sig2,UE_gam,...

m_l,m_nl,a_l,a_nl,K_l,K_nl,...
                                               m_I,a_I,K_I);
```

```
figure
hold
N=3;
plot(gam_db,P_BS_a,'r-');
plot(gam_db(1:N:end),P_BS_s(1:N:end),'rs');
plot(gam_db,P_UAV_a,'g-');
plot(gam_db(1:N:end),P_UAV_s(1:N:end),'gs')
plot(gam_db,P_NU_a,'b-');
plot(gam_db(1:N:end),P_NU_s(1:N:end),'bs');
```

- **Fig. 3: Network throughput vs. SINR threshold**

```
BS_I_lam=25e-6;
UE_lam=10000e-6;
KK_l=10.^(3./10);
KK_nl=10.^(23./10);
m_l=3;
m_nl=1;
a_l=2.5;
a_nl=4;
K_l=1./(KK_l.*(4.*pi.*2.*10.^9./(3.*10.^8)).^a_l);
K_nl=1./(KK_nl.*(4.*pi.*2.*10.^9./(3.*10.^8)).^a_nl);
P_UAV_UE=5;
H_UAV=300;
m_I=m_nl;
a_I=a_nl;
K_I=K_nl;
R_BS=100;
R_UAV=200;
R_0=300;
P_BS_UE=20;
P_BS_I=P_BS_UE;
N_cha=64;
UAV_cover_thet=pi./8;
sig2=10.^(-114./10);
gam_db=linspace(-30,30,100);
% gam_db=0;
UE_gam=10.^(gam_db./10);
% UE_gam=linspace(0,100,10)

[T_BS_a] =
R_BS_UE_T_ana(R_0,R_BS,R_UAV,H_UAV,UAV_cover_thet,N_cha,..
.
                                   UE_lam,BS_I_lam,...
```

```
                              P_BS_UE,P_BS_I,P_UAV_UE,...
                               sig2,UE_gam,...
                               
m_l,m_nl,a_l,a_nl,K_l,K_nl,...
                                             m_I,a_I,K_I);

[T_BS_s] = 
R_BS_UE_T_simu(R_0,R_BS,R_UAV,H_UAV,UAV_cover_thet,N_cha,.
..
                               UE_lam,BS_I_lam,...
                               P_BS_UE,P_BS_I,P_UAV_UE,...
                               sig2,UE_gam,...
                               
m_l,m_nl,a_l,a_nl,K_l,K_nl,...
                                             m_I,a_I,K_I);

[T_UAV_a] = 
R_UAV_UE_T_ana(R_0,R_BS,R_UAV,H_UAV,UAV_cover_thet,N_cha,.
..
                               UE_lam,BS_I_lam,...
                               P_BS_UE,P_BS_I,P_UAV_UE,...
                               sig2,UE_gam,...
                               
m_l,m_nl,a_l,a_nl,K_l,K_nl,...
                                             m_I,a_I,K_I);

[T_UAV_s] = 
R_UAV_UE_T_simu(R_0,R_BS,R_UAV,H_UAV,UAV_cover_thet,N_cha,
...
                               UE_lam,BS_I_lam,...
                               P_BS_UE,P_BS_I,P_UAV_UE,...
                               sig2,UE_gam,...
                               
m_l,m_nl,a_l,a_nl,K_l,K_nl,...
                                             m_I,a_I,K_I);

[T_NU_a] = 
NU_R_BS_UE_T_ana(R_0,R_BS,R_UAV,H_UAV,UAV_cover_thet,N_ch
a,...
                               UE_lam,BS_I_lam,...
                               P_BS_UE,P_BS_I,P_UAV_UE,...
                               sig2,UE_gam,...
                               
m_l,m_nl,a_l,a_nl,K_l,K_nl,...
```

```
                                                  m_I,a_I,K_I);

[T_NU_s] =
NU_R_BS_UE_T_simu(R_0,R_BS,R_UAV,H_UAV,UAV_cover_thet,N_c
ha,...
                                  UE_lam,BS_I_lam,...
                                  P_BS_UE,P_BS_I,P_UAV_UE,...
                                  sig2,UE_gam,...

m_l,m_nl,a_l,a_nl,K_l,K_nl,...
                                                  m_I,a_I,K_I);

figure
hold
N=3;
plot(gam_db,T_BS_a,'r-');
plot(gam_db(1:N:end),T_BS_s(1:N:end),'rs');
plot(gam_db,T_UAV_a,'g-');
plot(gam_db(1:N:end),T_UAV_s(1:N:end),'gs')
plot(gam_db,T_NU_a,'b-');
plot(gam_db(1:N:end),T_NU_s(1:N:end),'bs');
```

- **Fig. 4: Network throughput vs. UAV height**

```
BS_I_lam=25e-6;
UE_lam=10000e-6;
KK_l=10.^(3./10);
KK_nl=10.^(23./10);
m_l=3;
m_nl=1;
a_l=2.5;
a_nl=4;
K_l=1./(KK_l.*(4.*pi.*2.*10.^9./(3.*10.^8)).^a_l);
K_nl=1./(KK_nl.*(4.*pi.*2.*10.^9./(3.*10.^8)).^a_nl);
P_UAV_UE=5;
H_UAV=300;
m_I=m_nl;
a_I=a_nl;
K_I=K_nl;
R_BS=100;
R_UAV=200;
R_0=300;
P_BS_UE=20;
P_BS_I=P_BS_UE;
N_cha=64;
```

```
UAV_cover_thet=pi./8;
sig2=10.^(-114./10);
% gam_db=linspace(-30,30,500);
 gam_db=0;
UE_gam=10.^(gam_db./10);
% UE_gam=linspace(0,100,10)
H_UAV=0:10:1000;
NN=length(H_UAV);
T_BS_a_1=NaN(1,NN);
T_UAV_a_1=NaN(1,NN);
T_NU_a_1=NaN(1,NN);
T_UU_a_1=NaN(1,NN);
T_BS_a_2=NaN(1,NN);
T_UAV_a_2=NaN(1,NN);
T_NU_a_2=NaN(1,NN);
T_UU_a_2=NaN(1,NN);
T_BS_a_3=NaN(1,NN);
T_UAV_a_3=NaN(1,NN);
T_NU_a_3=NaN(1,NN);
T_UU_a_3=NaN(1,NN);
parfor i=1:NN
R_0=300;

[T_BS_a_1(i)] =
R_BS_UE_T_ana(R_0,R_BS,R_UAV,H_UAV(i),UAV_cover_thet,N_ch
a,...
                           UE_lam,BS_I_lam,...
                           P_BS_UE,P_BS_I,P_UAV_UE,...
                           sig2,UE_gam,...

m_l,m_nl,a_l,a_nl,K_l,K_nl,...
                                        m_I,a_I,K_I);

[T_UAV_a_1(i)] =
R_UAV_UE_T_ana(R_0,R_BS,R_UAV,H_UAV(i),UAV_cover_thet,N_c
ha,...
                           UE_lam,BS_I_lam,...
                           P_BS_UE,P_BS_I,P_UAV_UE,...
                           sig2,UE_gam,...

m_l,m_nl,a_l,a_nl,K_l,K_nl,...
                                        m_I,a_I,K_I);

[T_NU_a_1(i)] =
```

```
NU_R_BS_UE_T_ana(R_0,R_BS,R_UAV,H_UAV(i),UAV_cover_thet,N
_cha,...
                              UE_lam,BS_I_lam,...
                              P_BS_UE,P_BS_I,P_UAV_UE,...
                              sig2,UE_gam,...

m_l,m_nl,a_l,a_nl,K_l,K_nl,...
                                            m_I,a_I,K_I);


T_UU_a_1(i)=T_BS_a_1(i)+T_UAV_a_1(i);

R_0=500;

[T_BS_a_2(i)] =
R_BS_UE_T_ana(R_0,R_BS,R_UAV,H_UAV(i),UAV_cover_thet,N_ch
a,...
                              UE_lam,BS_I_lam,...
                              P_BS_UE,P_BS_I,P_UAV_UE,...
                              sig2,UE_gam,...

m_l,m_nl,a_l,a_nl,K_l,K_nl,...
                                            m_I,a_I,K_I);

[T_UAV_a_2(i)] =
R_UAV_UE_T_ana(R_0,R_BS,R_UAV,H_UAV(i),UAV_cover_thet,N_c
ha,...
                              UE_lam,BS_I_lam,...
                              P_BS_UE,P_BS_I,P_UAV_UE,...
                              sig2,UE_gam,...

m_l,m_nl,a_l,a_nl,K_l,K_nl,...
                                            m_I,a_I,K_I);

[T_NU_a_2(i)] =
NU_R_BS_UE_T_ana(R_0,R_BS,R_UAV,H_UAV(i),UAV_cover_thet,N
_cha,...
                              UE_lam,BS_I_lam,...
                              P_BS_UE,P_BS_I,P_UAV_UE,...
                              sig2,UE_gam,...

m_l,m_nl,a_l,a_nl,K_l,K_nl,...
                                            m_I,a_I,K_I);
```

```
T_UU_a_2(i)=T_BS_a_2(i)+T_UAV_a_2(i);

R_0=800;

[T_BS_a_3(i)] =
R_BS_UE_T_ana(R_0,R_BS,R_UAV,H_UAV(i),UAV_cover_thet,N_ch
a,...
                           UE_lam,BS_I_lam,...
                           P_BS_UE,P_BS_I,P_UAV_UE,...
                           sig2,UE_gam,...

m_l,m_nl,a_l,a_nl,K_l,K_nl,...
                                        m_I,a_I,K_I);

[T_UAV_a_3(i)] =
R_UAV_UE_T_ana(R_0,R_BS,R_UAV,H_UAV(i),UAV_cover_thet,N_c
ha,...
                           UE_lam,BS_I_lam,...
                           P_BS_UE,P_BS_I,P_UAV_UE,...
                           sig2,UE_gam,...

m_l,m_nl,a_l,a_nl,K_l,K_nl,...
                                        m_I,a_I,K_I);

[T_NU_a_3(i)] =
NU_R_BS_UE_T_ana(R_0,R_BS,R_UAV,H_UAV(i),UAV_cover_thet,N
_cha,...
                           UE_lam,BS_I_lam,...
                           P_BS_UE,P_BS_I,P_UAV_UE,...
                           sig2,UE_gam,...

m_l,m_nl,a_l,a_nl,K_l,K_nl,...
                                        m_I,a_I,K_I);


T_UU_a_3(i)=T_BS_a_3(i)+T_UAV_a_3(i);

i

end

Figure
hold
N=3;
```

```
plot(H_UAV,T_UU_a_1,'r-');
% plot(gam_db(1:N:end),T_D_s_1(1:N:end),'rs');
plot(H_UAV,T_NU_a_1,'r--');
% plot(gam_db(1:N:end),T_S_s_1(1:N:end),'gs')
% plot(gam_db(1:N:end),T_OU_s_1(1:N:end),'ks')
plot(H_UAV,T_UU_a_2,'b-');
plot(H_UAV,T_NU_a_2,'b--');
plot(H_UAV,T_UU_a_3,'k-');
plot(H_UAV,T_NU_a_3,'k--');
% plot(H_UAV,T_OU_a_3,'k-.');
legend('UU R_C=300m','NU R_C=300m',...
    'UU R_C=500m','NU R_C=500m',...
    'UU R_C=800m','NU R_C=800m');
```

- **Fig. 5: Network traversal rate vs. UAV height**

```
BS_I_lam=25e-6;
UE_lam=10000e-6;
KK_l=10.^(3./10);
KK_nl=10.^(23./10);
m_l=3;
m_nl=1;
a_l=2.5;
a_nl=4;
K_l=1./(KK_l.*(4.*pi.*2.*10.^9./(3.*10.^8)).^a_l);
K_nl=1./(KK_nl.*(4.*pi.*2.*10.^9./(3.*10.^8)).^a_nl);
P_UAV_UE=5;
H_UAV=300;
m_I=m_nl;
a_I=a_nl;
K_I=K_nl;
R_BS=100;
R_UAV=200;
R_0=300;
P_BS_UE=20;
P_BS_I=P_BS_UE;
N_cha=64;
UAV_cover_thet=pi./8;
sig2=10.^(-114./10);
% gam_db=linspace(-30,30,500);
gam_db=0;
UE_gam=10.^(gam_db./10);
% UE_gam=linspace(0,100,10)
H_UAV=0:10:1000;
NN=length(H_UAV);
```

```
C_BS_a_1=NaN(1,NN);
C_UAV_a_1=NaN(1,NN);
C_NU_a_1=NaN(1,NN);
C_UU_a_1=NaN(1,NN);
C_BS_a_2=NaN(1,NN);
C_UAV_a_2=NaN(1,NN);
C_NU_a_2=NaN(1,NN);
C_UU_a_2=NaN(1,NN);
C_BS_a_3=NaN(1,NN);
C_UAV_a_3=NaN(1,NN);
C_NU_a_3=NaN(1,NN);
C_UU_a_3=NaN(1,NN);
h=0.01;
parfor i=1:NN
R_0=300;

[C_BS_a_1(i)] =
C_R_BS_UE_T_ana(R_0,R_BS,R_UAV,H_UAV(i),UAV_cover_thet,N_
cha,...
                          UE_lam,BS_I_lam,...
                          P_BS_UE,P_BS_I,P_UAV_UE,...
                          sig2,h,...

m_l,m_nl,a_l,a_nl,K_l,K_nl,...
                                      m_I,a_I,K_I);

[C_UAV_a_1(i)] =
C_R_UAV_UE_T_ana(R_0,R_BS,R_UAV,H_UAV(i),UAV_cover_thet,N
_cha,...
                          UE_lam,BS_I_lam,...
                          P_BS_UE,P_BS_I,P_UAV_UE,...
                          sig2,h,...

m_l,m_nl,a_l,a_nl,K_l,K_nl,...
                                      m_I,a_I,K_I);

[C_NU_a_1(i)] =
NU_C_R_BS_UE_T_ana(R_0,R_BS,R_UAV,H_UAV(i),UAV_cover_thet,
N_cha,...
                          UE_lam,BS_I_lam,...
                          P_BS_UE,P_BS_I,P_UAV_UE,...
                          sig2,h,...

m_l,m_nl,a_l,a_nl,K_l,K_nl,...
```

```
                                       m_I,a_I,K_I);

C_UU_a_1(i)=C_BS_a_1(i)+C_UAV_a_1(i);

R_0=500;

[C_BS_a_2(i)] =
C_R_BS_UE_T_ana(R_0,R_BS,R_UAV,H_UAV(i),UAV_cover_thet,N_
cha,...
                           UE_lam,BS_I_lam,...
                           P_BS_UE,P_BS_I,P_UAV_UE,...
                           sig2,h,...

m_l,m_nl,a_l,a_nl,K_l,K_nl,...
                                       m_I,a_I,K_I);

[C_UAV_a_2(i)] =
C_R_UAV_UE_T_ana(R_0,R_BS,R_UAV,H_UAV(i),UAV_cover_thet,N
_cha,...
                           UE_lam,BS_I_lam,...
                           P_BS_UE,P_BS_I,P_UAV_UE,...
                           sig2,h,...

m_l,m_nl,a_l,a_nl,K_l,K_nl,...
                                       m_I,a_I,K_I);

[C_NU_a_2(i)] =
NU_C_R_BS_UE_T_ana(R_0,R_BS,R_UAV,H_UAV(i),UAV_cover_thet,
N_cha,...
                           UE_lam,BS_I_lam,...
                           P_BS_UE,P_BS_I,P_UAV_UE,...
                           sig2,h,...

m_l,m_nl,a_l,a_nl,K_l,K_nl,...
                                       m_I,a_I,K_I);


C_UU_a_2(i)=C_BS_a_2(i)+C_UAV_a_2(i);
R_0=800;

[C_BS_a_3(i)] =
C_R_BS_UE_T_ana(R_0,R_BS,R_UAV,H_UAV(i),UAV_cover_thet,N_
cha,...
                           UE_lam,BS_I_lam,...
```

```
                                P_BS_UE,P_BS_I,P_UAV_UE,...
                                sig2,h,...

m_l,m_nl,a_l,a_nl,K_l,K_nl,...
                                               m_I,a_I,K_I);

[C_UAV_a_3(i)] =
C_R_UAV_UE_T_ana(R_0,R_BS,R_UAV,H_UAV(i),UAV_cover_thet,N
_cha,...
                                UE_lam,BS_I_lam,...
                                P_BS_UE,P_BS_I,P_UAV_UE,...
                                sig2,h,...

m_l,m_nl,a_l,a_nl,K_l,K_nl,...
                                               m_I,a_I,K_I);

[C_NU_a_3(i)] =
NU_C_R_BS_UE_T_ana(R_0,R_BS,R_UAV,H_UAV(i),UAV_cover_thet,
N_cha,...
                                UE_lam,BS_I_lam,...
                                P_BS_UE,P_BS_I,P_UAV_UE,...
                                sig2,h,...

m_l,m_nl,a_l,a_nl,K_l,K_nl,...
                                               m_I,a_I,K_I);

C_UU_a_3(i)=C_BS_a_3(i)+C_UAV_a_3(i);

i

end

figure
hold
N=3;
plot(H_UAV,C_UU_a_1,'r-');
% plot(gam_db(1:N:end),T_D_s_1(1:N:end),'rs');
plot(H_UAV,C_NU_a_1,'r--');
% plot(gam_db(1:N:end),T_S_s_1(1:N:end),'gs')
% plot(gam_db(1:N:end),T_OU_s_1(1:N:end),'ks')
plot(H_UAV,C_UU_a_2,'b-');
plot(H_UAV,C_NU_a_2,'b--');
plot(H_UAV,C_UU_a_3,'k-');
plot(H_UAV,C_NU_a_3,'k--');
```

```
% plot(H_UAV,T_OU_a_3,'k-.');
legend('UU R_C=300m','NU R_C=300m',...
    'UU R_C=500m','NU R_C=500m',...
    'UU R_C=800m','NU R_C=800m');
```

- **Fig. 6: Network throughput vs. UAV radius**

```
BS_I_lam=25e-6;
UE_lam=10000e-6;
KK_l=10.^(3./10);
KK_nl=10.^(23./10);
m_l=3;
m_nl=1;
a_l=2.5;
a_nl=4;
K_l=1./(KK_l.*(4.*pi.*2.*10.^9./(3.*10.^8)).^a_l);
K_nl=1./(KK_nl.*(4.*pi.*2.*10.^9./(3.*10.^8)).^a_nl);
P_UAV_UE=5;
H_UAV=300;
m_I=m_nl;
a_I=a_nl;
K_I=K_nl;
R_BS=100;
R_UAV=200;
R_0=300;
P_BS_UE=20;
P_BS_I=P_BS_UE;
N_cha=64;
UAV_cover_thet=pi./8;
sig2=10.^(-114./10);
% gam_db=linspace(-30,30,500);
 gam_db=0;
UE_gam=10.^(gam_db./10);
% UE_gam=linspace(0,100,10)
R_UAV=0:10:1000;
N_bian=R_UAV;
NN=length(N_bian);
T_BS_a_1=NaN(1,NN);
T_UAV_a_1=NaN(1,NN);
T_NU_a_1=NaN(1,NN);
T_UU_a_1=NaN(1,NN);
T_BS_a_2=NaN(1,NN);
T_UAV_a_2=NaN(1,NN);
T_NU_a_2=NaN(1,NN);
T_UU_a_2=NaN(1,NN);
```

```
T_BS_a_3=NaN(1,NN);
T_UAV_a_3=NaN(1,NN);
T_NU_a_3=NaN(1,NN);
T_UU_a_3=NaN(1,NN);

parfor i=1:NN
R_0=300;

[T_BS_a_1(i)] =
R_BS_UE_T_ana(R_0,R_BS,R_UAV(i),H_UAV,UAV_cover_thet,N_ch
a,...
                          UE_lam,BS_I_lam,...
                          P_BS_UE,P_BS_I,P_UAV_UE,...
                          sig2,UE_gam,...

m_l,m_nl,a_l,a_nl,K_l,K_nl,...
                                      m_I,a_I,K_I);

[T_UAV_a_1(i)] =
R_UAV_UE_T_ana(R_0,R_BS,R_UAV(i),H_UAV,UAV_cover_thet,N_c
ha,...
                          UE_lam,BS_I_lam,...
                          P_BS_UE,P_BS_I,P_UAV_UE,...
                          sig2,UE_gam,...

m_l,m_nl,a_l,a_nl,K_l,K_nl,...
                                      m_I,a_I,K_I);

[T_NU_a_1(i)] =
NU_R_BS_UE_T_ana(R_0,R_BS,R_UAV(i),H_UAV,UAV_cover_thet,N
_cha,...
                          UE_lam,BS_I_lam,...
                          P_BS_UE,P_BS_I,P_UAV_UE,...
                          sig2,UE_gam,...

m_l,m_nl,a_l,a_nl,K_l,K_nl,...
                                      m_I,a_I,K_I);

T_UU_a_1(i)=T_BS_a_1(i)+T_UAV_a_1(i);

R_0=500;
[T_BS_a_2(i)] =
R_BS_UE_T_ana(R_0,R_BS,R_UAV(i),H_UAV,UAV_cover_thet,N_ch
a,...
```

```
                                UE_lam,BS_I_lam,...
                                P_BS_UE,P_BS_I,P_UAV_UE,...
                                sig2,UE_gam,...

m_l,m_nl,a_l,a_nl,K_l,K_nl,...
                                            m_I,a_I,K_I);

[T_UAV_a_2(i)] =
R_UAV_UE_T_ana(R_0,R_BS,R_UAV(i),H_UAV,UAV_cover_thet,N_c
ha,...
                                UE_lam,BS_I_lam,...
                                P_BS_UE,P_BS_I,P_UAV_UE,...
                                sig2,UE_gam,...

m_l,m_nl,a_l,a_nl,K_l,K_nl,...
                                            m_I,a_I,K_I);

[T_NU_a_2(i)] =
NU_R_BS_UE_T_ana(R_0,R_BS,R_UAV(i),H_UAV,UAV_cover_thet,N
_cha,...
                                UE_lam,BS_I_lam,...
                                P_BS_UE,P_BS_I,P_UAV_UE,...
                                sig2,UE_gam,...

m_l,m_nl,a_l,a_nl,K_l,K_nl,...
                                            m_I,a_I,K_I);

T_UU_a_2(i)=T_BS_a_2(i)+T_UAV_a_2(i);

R_0=800;

[T_BS_a_3(i)] =
R_BS_UE_T_ana(R_0,R_BS,R_UAV(i),H_UAV,UAV_cover_thet,N_ch
a,...
                                UE_lam,BS_I_lam,...
                                P_BS_UE,P_BS_I,P_UAV_UE,...
                                sig2,UE_gam,...

m_l,m_nl,a_l,a_nl,K_l,K_nl,...
                                            m_I,a_I,K_I);

[T_UAV_a_3(i)] =
R_UAV_UE_T_ana(R_0,R_BS,R_UAV(i),H_UAV,UAV_cover_thet,N_c
ha,...
```

```
                                UE_lam,BS_I_lam,...
                                P_BS_UE,P_BS_I,P_UAV_UE,...
                                sig2,UE_gam,...

m_l,m_nl,a_l,a_nl,K_l,K_nl,...
                                            m_I,a_I,K_I);

[T_NU_a_3(i)] =
NU_R_BS_UE_T_ana(R_0,R_BS,R_UAV(i),H_UAV,UAV_cover_thet,N
_cha,...
                                UE_lam,BS_I_lam,...
                                P_BS_UE,P_BS_I,P_UAV_UE,...
                                sig2,UE_gam,...

m_l,m_nl,a_l,a_nl,K_l,K_nl,...
                                            m_I,a_I,K_I);

T_UU_a_3(i)=T_BS_a_3(i)+T_UAV_a_3(i);

i

end

figure
hold
N=3;
plot(N_bian,T_UU_a_1,'r-');
plot(N_bian,T_NU_a_1,'r--');
plot(N_bian,T_UU_a_2,'b-');
plot(N_bian,T_NU_a_2,'b--');
plot(N_bian,T_UU_a_3,'k-');
plot(N_bian,T_NU_a_3,'k--');
legend('UU R_C=300m','NU R_C=300m',...
   'UU R_C=500m','NU R_C=500m',...
   'UU R_C=800m','NU R_C=800m');
```

- **Fig. 7: Network traversal rate vs. UAV radius**

```
BS_I_lam=25e-6;
UE_lam=10000e-6;
KK_l=10.^(3./10);
KK_nl=10.^(23./10);
m_l=3;
m_nl=1;
a_l=2.5;
```

```
a_nl=4;
K_l=1./(KK_l.*(4.*pi.*2.*10.^9./(3.*10.^8)).^a_l);
K_nl=1./(KK_nl.*(4.*pi.*2.*10.^9./(3.*10.^8)).^a_nl);
P_UAV_UE=5;
H_UAV=300;
m_I=m_nl;
a_I=a_nl;
K_I=K_nl;
R_BS=100;
R_UAV=200;
R_0=300;
P_BS_UE=20;
P_BS_I=P_BS_UE;
N_cha=64;
UAV_cover_thet=pi./8;
sig2=10.^(-114./10);
gam_db=0;
UE_gam=10.^(gam_db./10);
R_UAV=0:10:1000;
N_bian=R_UAV;
NN=length(R_UAV);
C_BS_a_1=NaN(1,NN);
C_UAV_a_1=NaN(1,NN);
C_NU_a_1=NaN(1,NN);
C_UU_a_1=NaN(1,NN);
C_BS_a_2=NaN(1,NN);
C_UAV_a_2=NaN(1,NN);
C_NU_a_2=NaN(1,NN);
C_UU_a_2=NaN(1,NN);
C_BS_a_3=NaN(1,NN);
C_UAV_a_3=NaN(1,NN);
C_NU_a_3=NaN(1,NN);
C_UU_a_3=NaN(1,NN);
h=0.01;
parfor i=1:NN
R_0=300;
[C_BS_a_1(i)] =
C_R_BS_UE_T_ana(R_0,R_BS,R_UAV(i),H_UAV,UAV_cover_thet,N_
cha,...
                           UE_lam,BS_I_lam,...
                           P_BS_UE,P_BS_I,P_UAV_UE,...
                           sig2,h,...

m_l,m_nl,a_l,a_nl,K_l,K_nl,...
```

```
                                           m_I,a_I,K_I);

[C_UAV_a_1(i)] =
C_R_UAV_UE_T_ana(R_0,R_BS,R_UAV(i),H_UAV,UAV_cover_thet,N
_cha,...
                           UE_lam,BS_I_lam,...
                           P_BS_UE,P_BS_I,P_UAV_UE,...
                           sig2,h,...

m_l,m_nl,a_l,a_nl,K_l,K_nl,...
                                           m_I,a_I,K_I);

[C_NU_a_1(i)] =
NU_C_R_BS_UE_T_ana(R_0,R_BS,R_UAV(i),H_UAV,UAV_cover_thet,
N_cha,...
                           UE_lam,BS_I_lam,...
                           P_BS_UE,P_BS_I,P_UAV_UE,...
                           sig2,h,...

m_l,m_nl,a_l,a_nl,K_l,K_nl,...
                                           m_I,a_I,K_I);

C_UU_a_1(i)=C_BS_a_1(i)+C_UAV_a_1(i);
R_0=500;

[C_BS_a_2(i)] =
C_R_BS_UE_T_ana(R_0,R_BS,R_UAV(i),H_UAV,UAV_cover_thet,N_
cha,...
                           UE_lam,BS_I_lam,...
                           P_BS_UE,P_BS_I,P_UAV_UE,...
                           sig2,h,...

m_l,m_nl,a_l,a_nl,K_l,K_nl,...
                                           m_I,a_I,K_I);

[C_UAV_a_2(i)] =
C_R_UAV_UE_T_ana(R_0,R_BS,R_UAV(i),H_UAV,UAV_cover_thet,N
_cha,...
                           UE_lam,BS_I_lam,...
                           P_BS_UE,P_BS_I,P_UAV_UE,...
                           sig2,h,...

m_l,m_nl,a_l,a_nl,K_l,K_nl,...
                                           m_I,a_I,K_I);
```

```
[C_NU_a_2(i)] =
NU_C_R_BS_UE_T_ana(R_0,R_BS,R_UAV(i),H_UAV,UAV_cover_thet,
N_cha,...
                          UE_lam,BS_I_lam,...
                          P_BS_UE,P_BS_I,P_UAV_UE,...
                          sig2,h,...

m_l,m_nl,a_l,a_nl,K_l,K_nl,...
                                      m_I,a_I,K_I);

C_UU_a_2(i)=C_BS_a_2(i)+C_UAV_a_2(i);

R_0=800;
[C_BS_a_3(i)] =
C_R_BS_UE_T_ana(R_0,R_BS,R_UAV(i),H_UAV,UAV_cover_thet,N_
cha,...
                          UE_lam,BS_I_lam,...
                          P_BS_UE,P_BS_I,P_UAV_UE,...
                          sig2,h,...

m_l,m_nl,a_l,a_nl,K_l,K_nl,...
                                      m_I,a_I,K_I);

[C_UAV_a_3(i)] =
C_R_UAV_UE_T_ana(R_0,R_BS,R_UAV(i),H_UAV,UAV_cover_thet,N
_cha,...
                          UE_lam,BS_I_lam,...
                          P_BS_UE,P_BS_I,P_UAV_UE,...
                          sig2,h,...

m_l,m_nl,a_l,a_nl,K_l,K_nl,...
                                      m_I,a_I,K_I);


[C_NU_a_3(i)] =
NU_C_R_BS_UE_T_ana(R_0,R_BS,R_UAV(i),H_UAV,UAV_cover_thet,
N_cha,...
                          UE_lam,BS_I_lam,...
                          P_BS_UE,P_BS_I,P_UAV_UE,...
                          sig2,h,...

m_l,m_nl,a_l,a_nl,K_l,K_nl,...
```

```
                                        m_I,a_I,K_I);

C_UU_a_3(i)=C_BS_a_3(i)+C_UAV_a_3(i);

i
end

figure
hold
N=3;
plot(N_bian,C_UU_a_1,'r-');
plot(N_bian,C_NU_a_1,'r--');
plot(N_bian,C_UU_a_2,'b-');
plot(N_bian,C_NU_a_2,'b--');
plot(N_bian,C_UU_a_3,'k-');
plot(N_bian,C_NU_a_3,'k--');
legend('UU R_C=300m','NU R_C=300m',...
    'UU R_C=500m','NU R_C=500m',...
    'UU R_C=800m','NU R_C=800m');
```

- **Fig. 8: Network throughput vs. UAV around arc**

```
BS_I_lam=25e-6;
UE_lam=10000e-6;
KK_l=10.^(3./10);
KK_nl=10.^(23./10);
m_l=3;
m_nl=1;
a_l=2.5;
a_nl=4;
K_l=1./(KK_l.*(4.*pi.*2.*10.^9./(3.*10.^8)).^a_l);
K_nl=1./(KK_nl.*(4.*pi.*2.*10.^9./(3.*10.^8)).^a_nl);
P_UAV_UE=5;
H_UAV=300;
m_I=m_nl;
a_I=a_nl;
K_I=K_nl;
R_BS=100;
R_UAV=200;
R_0=300;
P_BS_UE=20;
P_BS_I=P_BS_UE;
N_cha=64;
UAV_cover_thet=pi./8;
sig2=10.^(-114./10);
```

```
gam_db=0;
UE_gam=10.^(gam_db./10);
UAV_cover_thet=linspace(0,pi./5,201);
N_bian=UAV_cover_thet;
NN=length(N_bian);
T_BS_a_1=NaN(1,NN);
T_UAV_a_1=NaN(1,NN);
T_NU_a_1=NaN(1,NN);
T_UU_a_1=NaN(1,NN);
T_BS_a_2=NaN(1,NN);
T_UAV_a_2=NaN(1,NN);
T_NU_a_2=NaN(1,NN);
T_UU_a_2=NaN(1,NN);
T_BS_a_3=NaN(1,NN);
T_UAV_a_3=NaN(1,NN);
T_NU_a_3=NaN(1,NN);
T_UU_a_3=NaN(1,NN);

parfor i=1:NN
R_0=300;
[T_BS_a_1(i)] =
R_BS_UE_T_ana(R_0,R_BS,R_UAV,H_UAV,UAV_cover_thet(i),N_ch
a,...
                           UE_lam,BS_I_lam,...
                           P_BS_UE,P_BS_I,P_UAV_UE,...
                           sig2,UE_gam,...

m_l,m_nl,a_l,a_nl,K_l,K_nl,...
                                       m_I,a_I,K_I);

[T_UAV_a_1(i)] =
R_UAV_UE_T_ana(R_0,R_BS,R_UAV,H_UAV,UAV_cover_thet(i),N_c
ha,...
                           UE_lam,BS_I_lam,...
                           P_BS_UE,P_BS_I,P_UAV_UE,...
                           sig2,UE_gam,...

m_l,m_nl,a_l,a_nl,K_l,K_nl,...
                                       m_I,a_I,K_I);


[T_NU_a_1(i)] =
NU_R_BS_UE_T_ana(R_0,R_BS,R_UAV,H_UAV,UAV_cover_thet(i),N
_cha,...
```

```
                          UE_lam,BS_I_lam,...
                          P_BS_UE,P_BS_I,P_UAV_UE,...
                          sig2,UE_gam,...

m_l,m_nl,a_l,a_nl,K_l,K_nl,...
                                      m_I,a_I,K_I);


T_UU_a_1(i)=T_BS_a_1(i)+T_UAV_a_1(i);

R_0=500;

[T_BS_a_2(i)] =
R_BS_UE_T_ana(R_0,R_BS,R_UAV,H_UAV,UAV_cover_thet(i),N_ch
a,...
                          UE_lam,BS_I_lam,...
                          P_BS_UE,P_BS_I,P_UAV_UE,...
                          sig2,UE_gam,...

m_l,m_nl,a_l,a_nl,K_l,K_nl,...
                                      m_I,a_I,K_I);


[T_UAV_a_2(i)] =
R_UAV_UE_T_ana(R_0,R_BS,R_UAV,H_UAV,UAV_cover_thet(i),N_c
ha,...
                          UE_lam,BS_I_lam,...
                          P_BS_UE,P_BS_I,P_UAV_UE,...
                          sig2,UE_gam,...

m_l,m_nl,a_l,a_nl,K_l,K_nl,...
                                      m_I,a_I,K_I);


[T_NU_a_2(i)] =
NU_R_BS_UE_T_ana(R_0,R_BS,R_UAV,H_UAV,UAV_cover_thet(i),N
_cha,...
                          UE_lam,BS_I_lam,...
                          P_BS_UE,P_BS_I,P_UAV_UE,...
                          sig2,UE_gam,...

m_l,m_nl,a_l,a_nl,K_l,K_nl,...
                                      m_I,a_I,K_I);
```

```
T_UU_a_2(i)=T_BS_a_2(i)+T_UAV_a_2(i);


R_0=800;

[T_BS_a_3(i)] =
R_BS_UE_T_ana(R_0,R_BS,R_UAV,H_UAV,UAV_cover_thet(i),N_ch
a,...
                          UE_lam,BS_I_lam,...
                          P_BS_UE,P_BS_I,P_UAV_UE,...
                          sig2,UE_gam,...

m_l,m_nl,a_l,a_nl,K_l,K_nl,...
                                      m_I,a_I,K_I);


[T_UAV_a_3(i)] =
R_UAV_UE_T_ana(R_0,R_BS,R_UAV,H_UAV,UAV_cover_thet(i),N_c
ha,...
                          UE_lam,BS_I_lam,...
                          P_BS_UE,P_BS_I,P_UAV_UE,...
                          sig2,UE_gam,...

m_l,m_nl,a_l,a_nl,K_l,K_nl,...
                                      m_I,a_I,K_I);


[T_NU_a_3(i)] =
NU_R_BS_UE_T_ana(R_0,R_BS,R_UAV,H_UAV,UAV_cover_thet(i),N
_cha,...
                          UE_lam,BS_I_lam,...
                          P_BS_UE,P_BS_I,P_UAV_UE,...
                          sig2,UE_gam,...

m_l,m_nl,a_l,a_nl,K_l,K_nl,...
                                      m_I,a_I,K_I);


T_UU_a_3(i)=T_BS_a_3(i)+T_UAV_a_3(i);

i
end
```

```
figure
hold
N=3;
plot(N_bian,T_UU_a_1,'r-');
plot(N_bian,T_NU_a_1,'r--');
plot(N_bian,T_UU_a_2,'b-');
plot(N_bian,T_NU_a_2,'b--');
plot(N_bian,T_UU_a_3,'k-');
plot(N_bian,T_NU_a_3,'k--');
legend('UU R_C=300m','NU R_C=300m',...
    'UU R_C=500m','NU R_C=500m',...
    'UU R_C=800m','NU R_C=800m');
```

- **Fig. 9: Network traversal rate vs. UAV around arc**

```
BS_I_lam=25e-6;
UE_lam=10000e-6;
KK_l=10.^(3./10);
KK_nl=10.^(23./10);
m_l=3;
m_nl=1;
a_l=2.5;
a_nl=4;
K_l=1./(KK_l.*(4.*pi.*2.*10.^9./(3.*10.^8)).^a_l);
K_nl=1./(KK_nl.*(4.*pi.*2.*10.^9./(3.*10.^8)).^a_nl);
P_UAV_UE=5;
H_UAV=300;
m_I=m_nl;
a_I=a_nl;
K_I=K_nl;
R_BS=100;
R_UAV=200;
R_0=300;
P_BS_UE=20;
P_BS_I=P_BS_UE;
N_cha=64;
UAV_cover_thet=pi./8;
sig2=10.^(-114./10);
gam_db=0;
UE_gam=10.^(gam_db./10);
UAV_cover_thet=linspace(0,pi./5,201);
N_bian=UAV_cover_thet;
NN=length(N_bian);
C_BS_a_1=NaN(1,NN);
C_UAV_a_1=NaN(1,NN);
```

```
C_NU_a_1=NaN(1,NN);
C_UU_a_1=NaN(1,NN);
C_BS_a_2=NaN(1,NN);
C_UAV_a_2=NaN(1,NN);
C_NU_a_2=NaN(1,NN);
C_UU_a_2=NaN(1,NN);
C_BS_a_3=NaN(1,NN);
C_UAV_a_3=NaN(1,NN);
C_NU_a_3=NaN(1,NN);
C_UU_a_3=NaN(1,NN);
h=0.01;
parfor i=1:NN

R_0=300;
[C_BS_a_1(i)] =
C_R_BS_UE_T_ana(R_0,R_BS,R_UAV,H_UAV,UAV_cover_thet(i),N_
cha,...
                          UE_lam,BS_I_lam,...
                          P_BS_UE,P_BS_I,P_UAV_UE,...
                          sig2,h,...

m_l,m_nl,a_l,a_nl,K_l,K_nl,...
                                      m_I,a_I,K_I);

[C_UAV_a_1(i)] =
C_R_UAV_UE_T_ana(R_0,R_BS,R_UAV,H_UAV,UAV_cover_thet(i),N
_cha,...
                          UE_lam,BS_I_lam,...
                          P_BS_UE,P_BS_I,P_UAV_UE,...
                          sig2,h,...

m_l,m_nl,a_l,a_nl,K_l,K_nl,...
                                      m_I,a_I,K_I);


[C_NU_a_1(i)] =
NU_C_R_BS_UE_T_ana(R_0,R_BS,R_UAV,H_UAV,UAV_cover_thet(i),
N_cha,...
                          UE_lam,BS_I_lam,...
                          P_BS_UE,P_BS_I,P_UAV_UE,...
                          sig2,h,...

m_l,m_nl,a_l,a_nl,K_l,K_nl,...
                                      m_I,a_I,K_I);
```

```
C_UU_a_1(i)=C_BS_a_1(i)+C_UAV_a_1(i);


R_0=500;
[C_BS_a_2(i)] =
C_R_BS_UE_T_ana(R_0,R_BS,R_UAV,H_UAV,UAV_cover_thet(i),N_
cha,...
                          UE_lam,BS_I_lam,...
                          P_BS_UE,P_BS_I,P_UAV_UE,...
                          sig2,h,...

m_l,m_nl,a_l,a_nl,K_l,K_nl,...
                                      m_I,a_I,K_I);

[C_UAV_a_2(i)] =
C_R_UAV_UE_T_ana(R_0,R_BS,R_UAV,H_UAV,UAV_cover_thet(i),N
_cha,...
                          UE_lam,BS_I_lam,...
                          P_BS_UE,P_BS_I,P_UAV_UE,...
                          sig2,h,...

m_l,m_nl,a_l,a_nl,K_l,K_nl,...
                                      m_I,a_I,K_I);


[C_NU_a_2(i)] =
NU_C_R_BS_UE_T_ana(R_0,R_BS,R_UAV,H_UAV,UAV_cover_thet(i),
N_cha,...
                          UE_lam,BS_I_lam,...
                          P_BS_UE,P_BS_I,P_UAV_UE,...
                          sig2,h,...

m_l,m_nl,a_l,a_nl,K_l,K_nl,...
                                      m_I,a_I,K_I);

 C_UU_a_2(i)=C_BS_a_2(i)+C_UAV_a_2(i);

R_0=800;
[C_BS_a_3(i)] =
C_R_BS_UE_T_ana(R_0,R_BS,R_UAV,H_UAV,UAV_cover_thet(i),N_
cha,...
                          UE_lam,BS_I_lam,...
```

```
                                 P_BS_UE,P_BS_I,P_UAV_UE,...
                                 sig2,h,...

m_l,m_nl,a_l,a_nl,K_l,K_nl,...
                                                m_I,a_I,K_I);

[C_UAV_a_3(i)] =
C_R_UAV_UE_T_ana(R_0,R_BS,R_UAV,H_UAV,UAV_cover_thet(i),N
_cha,...
                                 UE_lam,BS_I_lam,...
                                 P_BS_UE,P_BS_I,P_UAV_UE,...
                                 sig2,h,...

m_l,m_nl,a_l,a_nl,K_l,K_nl,...
                                                m_I,a_I,K_I);


[C_NU_a_3(i)] =
NU_C_R_BS_UE_T_ana(R_0,R_BS,R_UAV,H_UAV,UAV_cover_thet(i),
N_cha,...
                                 UE_lam,BS_I_lam,...
                                 P_BS_UE,P_BS_I,P_UAV_UE,...
                                 sig2,h,...

m_l,m_nl,a_l,a_nl,K_l,K_nl,...
                                                m_I,a_I,K_I);


C_UU_a_3(i)=C_BS_a_3(i)+C_UAV_a_3(i);

i
End

figure
hold
N=3;
plot(N_bian,C_UU_a_1,'r-');
plot(N_bian,C_NU_a_1,'r--');
plot(N_bian,C_UU_a_2,'b-');
plot(N_bian,C_NU_a_2,'b--');
plot(N_bian,C_UU_a_3,'k-');
plot(N_bian,C_NU_a_3,'k--');
legend('UU R_C=300m','NU R_C=300m',...
    'UU R_C=500m','NU R_C=500m',...
```

```
    'UU R_C=800m','NU R_C=800m');
```

- **Fig. 10: Network throughput vs. Number of channel**

```
BS_I_lam=25e-6;
UE_lam=10000e-6;
KK_l=10.^(3./10);
KK_nl=10.^(23./10);
m_l=3;
m_nl=1;
a_l=2.5;
a_nl=4;
K_l=1./(KK_l.*(4.*pi.*2.*10.^9./(3.*10.^8)).^a_l);
K_nl=1./(KK_nl.*(4.*pi.*2.*10.^9./(3.*10.^8)).^a_nl);
P_UAV_UE=5;
H_UAV=300;
m_I=m_nl;
a_I=a_nl;
K_I=K_nl;
R_BS=100;
R_UAV=200;
R_0=300;
P_BS_UE=20;
P_BS_I=P_BS_UE;
N_cha=64;
UAV_cover_thet=pi./8;
sig2=10.^(-114./10);
gam_db=0;
UE_gam=10.^(gam_db./10);
N_cha=1:20:601;
N_bian=N_cha;
NN=length(N_bian);
T_BS_a_1=NaN(1,NN);
T_UAV_a_1=NaN(1,NN);
T_NU_a_1=NaN(1,NN);
T_UU_a_1=NaN(1,NN);
T_BS_a_2=NaN(1,NN);
T_UAV_a_2=NaN(1,NN);
T_NU_a_2=NaN(1,NN);
T_UU_a_2=NaN(1,NN);
T_BS_a_3=NaN(1,NN);
T_UAV_a_3=NaN(1,NN);
T_NU_a_3=NaN(1,NN);
T_UU_a_3=NaN(1,NN);
```

```
parfor i=1:NN

R_0=300;
[T_BS_a_1(i)] =
R_BS_UE_T_ana(R_0,R_BS,R_UAV,H_UAV,UAV_cover_thet,N_cha(i)
,...
                          UE_lam,BS_I_lam,...
                          P_BS_UE,P_BS_I,P_UAV_UE,...
                          sig2,UE_gam,...

m_l,m_nl,a_l,a_nl,K_l,K_nl,...
                                      m_I,a_I,K_I);

[T_UAV_a_1(i)] =
R_UAV_UE_T_ana(R_0,R_BS,R_UAV,H_UAV,UAV_cover_thet,N_cha(
i),...
                          UE_lam,BS_I_lam,...
                          P_BS_UE,P_BS_I,P_UAV_UE,...
                          sig2,UE_gam,...

m_l,m_nl,a_l,a_nl,K_l,K_nl,...
                                      m_I,a_I,K_I);


[T_NU_a_1(i)] =
NU_R_BS_UE_T_ana(R_0,R_BS,R_UAV,H_UAV,UAV_cover_thet,N_ch
a(i),...
                          UE_lam,BS_I_lam,...
                          P_BS_UE,P_BS_I,P_UAV_UE,...
                          sig2,UE_gam,...

m_l,m_nl,a_l,a_nl,K_l,K_nl,...
                                      m_I,a_I,K_I);


T_UU_a_1(i)=T_BS_a_1(i)+T_UAV_a_1(i);


R_0=500;
[T_BS_a_2(i)] =
R_BS_UE_T_ana(R_0,R_BS,R_UAV,H_UAV,UAV_cover_thet,N_cha(i)
,...
                          UE_lam,BS_I_lam,...
                          P_BS_UE,P_BS_I,P_UAV_UE,...
```

```
                           sig2,UE_gam,...

m_l,m_nl,a_l,a_nl,K_l,K_nl,...
                                       m_I,a_I,K_I);



[T_UAV_a_2(i)] =
R_UAV_UE_T_ana(R_0,R_BS,R_UAV,H_UAV,UAV_cover_thet,N_cha(
i),...
                           UE_lam,BS_I_lam,...
                           P_BS_UE,P_BS_I,P_UAV_UE,...
                           sig2,UE_gam,...

m_l,m_nl,a_l,a_nl,K_l,K_nl,...
                                       m_I,a_I,K_I);


[T_NU_a_2(i)] =
NU_R_BS_UE_T_ana(R_0,R_BS,R_UAV,H_UAV,UAV_cover_thet,N_ch
a(i),...
                           UE_lam,BS_I_lam,...
                           P_BS_UE,P_BS_I,P_UAV_UE,...
                           sig2,UE_gam,...

m_l,m_nl,a_l,a_nl,K_l,K_nl,...
                                       m_I,a_I,K_I);


T_UU_a_2(i)=T_BS_a_2(i)+T_UAV_a_2(i);


R_0=800;
[T_BS_a_3(i)] =
R_BS_UE_T_ana(R_0,R_BS,R_UAV,H_UAV,UAV_cover_thet,N_cha(i)
,...
                           UE_lam,BS_I_lam,...
                           P_BS_UE,P_BS_I,P_UAV_UE,...
                           sig2,UE_gam,...

m_l,m_nl,a_l,a_nl,K_l,K_nl,...
                                       m_I,a_I,K_I);
```

```
[T_UAV_a_3(i)] = 
R_UAV_UE_T_ana(R_0,R_BS,R_UAV,H_UAV,UAV_cover_thet,N_cha(
i),...
                           UE_lam,BS_I_lam,...
                           P_BS_UE,P_BS_I,P_UAV_UE,...
                           sig2,UE_gam,...
                           
m_l,m_nl,a_l,a_nl,K_l,K_nl,...
                                       m_I,a_I,K_I);


[T_NU_a_3(i)] = 
NU_R_BS_UE_T_ana(R_0,R_BS,R_UAV,H_UAV,UAV_cover_thet,N_ch
a(i),...
                           UE_lam,BS_I_lam,...
                           P_BS_UE,P_BS_I,P_UAV_UE,...
                           sig2,UE_gam,...
                           
m_l,m_nl,a_l,a_nl,K_l,K_nl,...
                                       m_I,a_I,K_I);


T_UU_a_3(i)=T_BS_a_3(i)+T_UAV_a_3(i);

i

end

figure
hold
N=3;
plot(N_bian,T_UU_a_1,'r-');
plot(N_bian,T_NU_a_1,'r--');
plot(N_bian,T_UU_a_2,'b-');
plot(N_bian,T_NU_a_2,'b--');
plot(N_bian,T_UU_a_3,'k-');
plot(N_bian,T_NU_a_3,'k--');
legend('UU R_C=300m','NU R_C=300m',...
    'UU R_C=500m','NU R_C=500m',...
    'UU R_C=800m','NU R_C=800m');
```

- **Fig. 11: Network traversal rate vs. Number of channel**

```
BS_I_lam=25e-6;
UE_lam=10000e-6;
KK_l=10.^(3./10);
KK_nl=10.^(23./10);
m_l=3;
m_nl=1;
a_l=2.5;
a_nl=4;
K_l=1./(KK_l.*(4.*pi.*2.*10.^9./(3.*10.^8)).^a_l);
K_nl=1./(KK_nl.*(4.*pi.*2.*10.^9./(3.*10.^8)).^a_nl);
P_UAV_UE=5;
H_UAV=300;
m_I=m_nl;
a_I=a_nl;
K_I=K_nl;
R_BS=100;
R_UAV=200;
R_0=300;
P_BS_UE=20;
P_BS_I=P_BS_UE;
N_cha=64;
UAV_cover_thet=pi./8;
sig2=10.^(-114./10);
gam_db=0;
UE_gam=10.^(gam_db./10);
N_cha=1:10:601;
N_bian=N_cha;
NN=length(N_bian);
C_BS_a_1=NaN(1,NN);
C_UAV_a_1=NaN(1,NN);
C_NU_a_1=NaN(1,NN);
C_UU_a_1=NaN(1,NN);
C_BS_a_2=NaN(1,NN);
C_UAV_a_2=NaN(1,NN);
C_NU_a_2=NaN(1,NN);
C_UU_a_2=NaN(1,NN);
C_BS_a_3=NaN(1,NN);
C_UAV_a_3=NaN(1,NN);
C_NU_a_3=NaN(1,NN);
C_UU_a_3=NaN(1,NN);
h=0.01;
parfor i=1:NN
```

```
R_0=300;
[C_BS_a_1(i)] =
C_R_BS_UE_T_ana(R_0,R_BS,R_UAV,H_UAV,UAV_cover_thet,N_cha
(i),...
                           UE_lam,BS_I_lam,...
                           P_BS_UE,P_BS_I,P_UAV_UE,...
                           sig2,h,...

m_l,m_nl,a_l,a_nl,K_l,K_nl,...
                                       m_I,a_I,K_I);


[C_UAV_a_1(i)] =
C_R_UAV_UE_T_ana(R_0,R_BS,R_UAV,H_UAV,UAV_cover_thet,N_ch
a(i),...
                           UE_lam,BS_I_lam,...
                           P_BS_UE,P_BS_I,P_UAV_UE,...
                           sig2,h,...

m_l,m_nl,a_l,a_nl,K_l,K_nl,...
                                       m_I,a_I,K_I);


[C_NU_a_1(i)] =
NU_C_R_BS_UE_T_ana(R_0,R_BS,R_UAV,H_UAV,UAV_cover_thet,N_
cha(i),...
                           UE_lam,BS_I_lam,...
                           P_BS_UE,P_BS_I,P_UAV_UE,...
                           sig2,h,...

m_l,m_nl,a_l,a_nl,K_l,K_nl,...
                                       m_I,a_I,K_I);


C_UU_a_1(i)=C_BS_a_1(i)+C_UAV_a_1(i);

R_0=500;
[C_BS_a_2(i)] =
C_R_BS_UE_T_ana(R_0,R_BS,R_UAV,H_UAV,UAV_cover_thet,N_cha
(i),...
                           UE_lam,BS_I_lam,...
                           P_BS_UE,P_BS_I,P_UAV_UE,...
                           sig2,h,...
```

```
m_l,m_nl,a_l,a_nl,K_l,K_nl,...
                                            m_I,a_I,K_I);

[C_UAV_a_2(i)] =
C_R_UAV_UE_T_ana(R_0,R_BS,R_UAV,H_UAV,UAV_cover_thet,N_ch
a(i),...
                          UE_lam,BS_I_lam,...
                          P_BS_UE,P_BS_I,P_UAV_UE,...
                          sig2,h,...

m_l,m_nl,a_l,a_nl,K_l,K_nl,...
                                            m_I,a_I,K_I);

[C_NU_a_2(i)] =
NU_C_R_BS_UE_T_ana(R_0,R_BS,R_UAV,H_UAV,UAV_cover_thet,N_
cha(i),...
                          UE_lam,BS_I_lam,...
                          P_BS_UE,P_BS_I,P_UAV_UE,...
                          sig2,h,...

m_l,m_nl,a_l,a_nl,K_l,K_nl,...
                                            m_I,a_I,K_I);


C_UU_a_2(i)=C_BS_a_2(i)+C_UAV_a_2(i);


R_0=800;
[C_BS_a_3(i)] =
C_R_BS_UE_T_ana(R_0,R_BS,R_UAV,H_UAV,UAV_cover_thet,N_cha
(i),...
                          UE_lam,BS_I_lam,...
                          P_BS_UE,P_BS_I,P_UAV_UE,...
                          sig2,h,...

m_l,m_nl,a_l,a_nl,K_l,K_nl,...
                                            m_I,a_I,K_I);

[C_UAV_a_3(i)] =
C_R_UAV_UE_T_ana(R_0,R_BS,R_UAV,H_UAV,UAV_cover_thet,N_ch
a(i),...
                          UE_lam,BS_I_lam,...
                          P_BS_UE,P_BS_I,P_UAV_UE,...
```

```
                                sig2,h,...
m_l,m_nl,a_l,a_nl,K_l,K_nl,...
                                               m_I,a_I,K_I);


[C_NU_a_3(i)] =
NU_C_R_BS_UE_T_ana(R_0,R_BS,R_UAV,H_UAV,UAV_cover_thet,N_
cha(i),...
                                UE_lam,BS_I_lam,...
                                P_BS_UE,P_BS_I,P_UAV_UE,...
                                sig2,h,...
m_l,m_nl,a_l,a_nl,K_l,K_nl,...
                                               m_I,a_I,K_I);


C_UU_a_3(i)=C_BS_a_3(i)+C_UAV_a_3(i);

i
end

figure
hold
N=3;
plot(N_bian,C_UU_a_1,'r-');
plot(N_bian,C_NU_a_1,'r--');
plot(N_bian,C_UU_a_2,'b-');
plot(N_bian,C_NU_a_2,'b--');
plot(N_bian,C_UU_a_3,'k-');
plot(N_bian,C_NU_a_3,'k--');
legend('UU R_C=300m','NU R_C=300m',...
    'UU R_C=500m','NU R_C=500m',...
    'UU R_C=800m','NU R_C=800m');
```

- **Fig. 12 Network throughput vs. User density**

```
BS_I_lam=25e-6;
UE_lam=10000e-6;
KK_l=10.^(3./10);
KK_nl=10.^(23./10);
m_l=3;
m_nl=1;
a_l=2.5;
a_nl=4;
```

```
K_l=1./(KK_l.*(4.*pi.*2.*10.^9./(3.*10.^8)).^a_l);
K_nl=1./(KK_nl.*(4.*pi.*2.*10.^9./(3.*10.^8)).^a_nl);
P_UAV_UE=5;
H_UAV=300;
m_I=m_nl;
a_I=a_nl;
K_I=K_nl;
R_BS=100;
R_UAV=200;
R_0=300;
P_BS_UE=20;
P_BS_I=P_BS_UE;
N_cha=64;
UAV_cover_thet=pi./8;
sig2=10.^(-114./10);
gam_db=0;
UE_gam=10.^(gam_db./10);
UE_lam=(0:100:10000).*1e-6;
N_bian=UE_lam;
NN=length(N_bian);
T_BS_a_1=NaN(1,NN);
T_UAV_a_1=NaN(1,NN);
T_NU_a_1=NaN(1,NN);
T_UU_a_1=NaN(1,NN);
T_BS_a_2=NaN(1,NN);
T_UAV_a_2=NaN(1,NN);
T_NU_a_2=NaN(1,NN);
T_UU_a_2=NaN(1,NN);
T_BS_a_3=NaN(1,NN);
T_UAV_a_3=NaN(1,NN);
T_NU_a_3=NaN(1,NN);
T_UU_a_3=NaN(1,NN);

parfor i=1:NN

R_0=300;
[T_BS_a_1(i)] =
R_BS_UE_T_ana(R_0,R_BS,R_UAV,H_UAV,UAV_cover_thet,N_cha,..
.
                           UE_lam(i),BS_I_lam,...
                           P_BS_UE,P_BS_I,P_UAV_UE,...
                           sig2,UE_gam,...

m_l,m_nl,a_l,a_nl,K_l,K_nl,...
```

```
                                m_I,a_I,K_I);


[T_UAV_a_1(i)] =
R_UAV_UE_T_ana(R_0,R_BS,R_UAV,H_UAV,UAV_cover_thet,N_cha,.
..
                          UE_lam(i),BS_I_lam,...
                          P_BS_UE,P_BS_I,P_UAV_UE,...
                          sig2,UE_gam,...

m_l,m_nl,a_l,a_nl,K_l,K_nl,...
                                m_I,a_I,K_I);
[T_NU_a_1(i)] =
NU_R_BS_UE_T_ana(R_0,R_BS,R_UAV,H_UAV,UAV_cover_thet,N_ch
a,...
                          UE_lam(i),BS_I_lam,...
                          P_BS_UE,P_BS_I,P_UAV_UE,...
                          sig2,UE_gam,...

m_l,m_nl,a_l,a_nl,K_l,K_nl,...
                                m_I,a_I,K_I);


T_UU_a_1(i)=T_BS_a_1(i)+T_UAV_a_1(i);


R_0=500;
[T_BS_a_2(i)] =
R_BS_UE_T_ana(R_0,R_BS,R_UAV,H_UAV,UAV_cover_thet,N_cha,..
.
                          UE_lam(i),BS_I_lam,...
                          P_BS_UE,P_BS_I,P_UAV_UE,...
                          sig2,UE_gam,...

m_l,m_nl,a_l,a_nl,K_l,K_nl,...
                                m_I,a_I,K_I);

[T_UAV_a_2(i)] =
R_UAV_UE_T_ana(R_0,R_BS,R_UAV,H_UAV,UAV_cover_thet,N_cha,.
..
                          UE_lam(i),BS_I_lam,...
                          P_BS_UE,P_BS_I,P_UAV_UE,...
                          sig2,UE_gam,...
```

```
m_l,m_nl,a_l,a_nl,K_l,K_nl,...
                                        m_I,a_I,K_I);

[T_NU_a_2(i)] =
NU_R_BS_UE_T_ana(R_0,R_BS,R_UAV,H_UAV,UAV_cover_thet,N_ch
a,...
                           UE_lam(i),BS_I_lam,...
                           P_BS_UE,P_BS_I,P_UAV_UE,...
                           sig2,UE_gam,...

m_l,m_nl,a_l,a_nl,K_l,K_nl,...
                                        m_I,a_I,K_I);


T_UU_a_2(i)=T_BS_a_2(i)+T_UAV_a_2(i);

R_0=800;
[T_BS_a_3(i)] =
R_BS_UE_T_ana(R_0,R_BS,R_UAV,H_UAV,UAV_cover_thet,N_cha,..
.
                           UE_lam(i),BS_I_lam,...
                           P_BS_UE,P_BS_I,P_UAV_UE,...
                           sig2,UE_gam,...

m_l,m_nl,a_l,a_nl,K_l,K_nl,...
                                        m_I,a_I,K_I);

[T_UAV_a_3(i)] =
R_UAV_UE_T_ana(R_0,R_BS,R_UAV,H_UAV,UAV_cover_thet,N_cha,.
..
                           UE_lam(i),BS_I_lam,...
                           P_BS_UE,P_BS_I,P_UAV_UE,...
                           sig2,UE_gam,...

m_l,m_nl,a_l,a_nl,K_l,K_nl,...
                                        m_I,a_I,K_I);


[T_NU_a_3(i)] =
NU_R_BS_UE_T_ana(R_0,R_BS,R_UAV,H_UAV,UAV_cover_thet,N_ch
a,...
                           UE_lam(i),BS_I_lam,...
                           P_BS_UE,P_BS_I,P_UAV_UE,...
                           sig2,UE_gam,...
```

```
m_l,m_nl,a_l,a_nl,K_l,K_nl,...
                                        m_I,a_I,K_I);


T_UU_a_3(i)=T_BS_a_3(i)+T_UAV_a_3(i);

i
end

figure
hold
N=3;
plot(N_bian,T_UU_a_1,'r-');
plot(N_bian,T_NU_a_1,'r--');
plot(N_bian,T_UU_a_2,'b-');
plot(N_bian,T_NU_a_2,'b--');
plot(N_bian,T_UU_a_3,'k-');
plot(N_bian,T_NU_a_3,'k--');
legend('UU R_C=300m','NU R_C=300m',...
    'UU R_C=500m','NU R_C=500m',...
    'UU R_C=800m','NU R_C=800m');
```

- **Fig. 13: Network traversal rate vs. User density**

```
BS_I_lam=25e-6;
UE_lam=10000e-6;
KK_l=10.^(3./10);
KK_nl=10.^(23./10);
m_l=3;
m_nl=1;
a_l=2.5;
a_nl=4;
K_l=1./(KK_l.*(4.*pi.*2.*10.^9./(3.*10.^8)).^a_l);
K_nl=1./(KK_nl.*(4.*pi.*2.*10.^9./(3.*10.^8)).^a_nl);
P_UAV_UE=5;
H_UAV=300;
m_I=m_nl;
a_I=a_nl;
K_I=K_nl;
R_BS=100;
R_UAV=200;
R_0=300;
P_BS_UE=20;
P_BS_I=P_BS_UE;
```

```
N_cha=64;
UAV_cover_thet=pi./8;
sig2=10.^(-114./10);
gam_db=0;
UE_gam=10.^(gam_db./10);
UE_lam=(0:100:10000).*1e-6;
N_bian=UE_lam;
NN=length(N_bian);
C_BS_a_1=NaN(1,NN);
C_UAV_a_1=NaN(1,NN);
C_NU_a_1=NaN(1,NN);
C_UU_a_1=NaN(1,NN);
C_BS_a_2=NaN(1,NN);
C_UAV_a_2=NaN(1,NN);
C_NU_a_2=NaN(1,NN);
C_UU_a_2=NaN(1,NN);
C_BS_a_3=NaN(1,NN);
C_UAV_a_3=NaN(1,NN);
C_NU_a_3=NaN(1,NN);
C_UU_a_3=NaN(1,NN);

h=0.01;
parfor i=1:NN

R_0=300;
[C_BS_a_1(i)] =
C_R_BS_UE_T_ana(R_0,R_BS,R_UAV,H_UAV,UAV_cover_thet,N
_cha,...
                           UE_lam(i),BS_I_lam,...

P_BS_UE,P_BS_I,P_UAV_UE,...
                           sig2,h,...

m_l,m_nl,a_l,a_nl,K_l,K_nl,...
                                        m_I,a_I,K_I);
[C_UAV_a_1(i)] =
C_R_UAV_UE_T_ana(R_0,R_BS,R_UAV,H_UAV,UAV_cover_thet,
N_cha,...
                           UE_lam(i),BS_I_lam,...

P_BS_UE,P_BS_I,P_UAV_UE,...
                           sig2,h,...
```

```
m_l,m_nl,a_l,a_nl,K_l,K_nl,...
                                        m_I,a_I,K_I);

[C_NU_a_1(i)] =
NU_C_R_BS_UE_T_ana(R_0,R_BS,R_UAV,H_UAV,UAV_cover_the
t,N_cha,...
                           UE_lam(i),BS_I_lam,...

P_BS_UE,P_BS_I,P_UAV_UE,...
                           sig2,h,...

m_l,m_nl,a_l,a_nl,K_l,K_nl,...
                                        m_I,a_I,K_I);

C_UU_a_1(i)=C_BS_a_1(i)+C_UAV_a_1(i);

R_0=500;

[C_BS_a_2(i)] =
C_R_BS_UE_T_ana(R_0,R_BS,R_UAV,H_UAV,UAV_cover_thet,N
_cha,...
                           UE_lam(i),BS_I_lam,...

P_BS_UE,P_BS_I,P_UAV_UE,...
                           sig2,h,...

m_l,m_nl,a_l,a_nl,K_l,K_nl,...
                                        m_I,a_I,K_I);

[C_UAV_a_2(i)] =
C_R_UAV_UE_T_ana(R_0,R_BS,R_UAV,H_UAV,UAV_cover_thet,
N_cha,...
                           UE_lam(i),BS_I_lam,...

P_BS_UE,P_BS_I,P_UAV_UE,...
                           sig2,h,...

m_l,m_nl,a_l,a_nl,K_l,K_nl,...
                                        m_I,a_I,K_I);

[C_NU_a_2(i)] =
NU_C_R_BS_UE_T_ana(R_0,R_BS,R_UAV,H_UAV,UAV_cover_the
t,N_cha,...
                           UE_lam(i),BS_I_lam,...
```

```
P_BS_UE,P_BS_I,P_UAV_UE,...
                           sig2,h,...

m_l,m_nl,a_l,a_nl,K_l,K_nl,...
                                        m_I,a_I,K_I);

C_UU_a_2(i)=C_BS_a_2(i)+C_UAV_a_2(i);

R_0=800;
[C_BS_a_3(i)] =
C_R_BS_UE_T_ana(R_0,R_BS,R_UAV,H_UAV,UAV_cover_thet,N
_cha,...
                           UE_lam(i),BS_I_lam,...

P_BS_UE,P_BS_I,P_UAV_UE,...
                           sig2,h,...

m_l,m_nl,a_l,a_nl,K_l,K_nl,...
                                        m_I,a_I,K_I);

[C_UAV_a_3(i)] =
C_R_UAV_UE_T_ana(R_0,R_BS,R_UAV,H_UAV,UAV_cover_thet,
N_cha,...
                           UE_lam(i),BS_I_lam,...

P_BS_UE,P_BS_I,P_UAV_UE,...
                           sig2,h,...

m_l,m_nl,a_l,a_nl,K_l,K_nl,...
                                        m_I,a_I,K_I);


[C_NU_a_3(i)] =
NU_C_R_BS_UE_T_ana(R_0,R_BS,R_UAV,H_UAV,UAV_cover_the
t,N_cha,...
                           UE_lam(i),BS_I_lam,...

P_BS_UE,P_BS_I,P_UAV_UE,...
                           sig2,h,...

m_l,m_nl,a_l,a_nl,K_l,K_nl,...
                                        m_I,a_I,K_I);
```

```
C_UU_a_3(i)=C_BS_a_3(i)+C_UAV_a_3(i);

i

end

figure
hold
N=3;
plot(N_bian,C_UU_a_1,'r-');
plot(N_bian,C_NU_a_1,'r--');
plot(N_bian,C_UU_a_2,'b-');
plot(N_bian,C_NU_a_2,'b--');
plot(N_bian,C_UU_a_3,'k-');
plot(N_bian,C_NU_a_3,'k--');
legend('UU R_C=300m','NU R_C=300m',...
    'UU R_C=500m','NU R_C=500m',...
    'UU R_C=800m','NU R_C=800m');
```
